# Tunable Reversible Photochromic Ultralong Organic Phosphorescence via a Universal Phenylpyridine Noncovalent Assembly Strategy

**DOI:** 10.1002/advs.76554

**Published:** 2026-07-21

**Authors:** Xue Bai, Renliang Wang, Hong‐Jin Xue, Zhuofan Jiang, Shuo Yang, Chiming Li, Yanqing Ge, Caihong Liu, Xian‐Yin Dai

**Affiliations:** ^1^ School of Chemistry and Pharmaceutical Engineering Shandong First Medical University & Shandong Academy of Medical Sciences Taian China; ^2^ Medical Engineering and Technology Research Center, School of Radiology Shandong First Medical University & Shandong Academy of Medical Sciences Jinan Jinan China; ^3^ School of Pharmaceutical Sciences & Institute of Materia Medica Shandong First Medical University & Shandong Academy of Medical Sciences Jinan China

**Keywords:** cyclodextrin, noncovalent assembly, photochromic, photoinduced radical, ultralong organic phosphorescence

## Abstract

Supramolecular photochromic materials capable of reversible alteration in visual color and photoluminescence upon light stimulation represent a pivotal frontier in contemporary materials science. Herein, we present a facile yet universal supramolecular assembly strategy based on linear phenylpyridine derivatives and α‐cyclodextrin, enabling the simultaneous switchable modulation of photochromism and ultralong organic phosphorescence (UOP) in the solid state. Multivalent interactions, including host‐guest complexation and hydrogen bonding, synergistically construct a robust noncovalent network that not only stabilizes excited triplet states with a maximum phosphorescence lifetime of 0.96 s and quantum yield of 53.1%, but also promotes efficient photoinduced electron transfer for in situ generation of stable radicals governing reversible photochromism. Notably, the UOP can be dynamically regulated via a photoinduced radical‐mediated photochromic switch. Furthermore, precision engineering of aryl substituents or pyridine substitution sites on guest molecules enables tailorable phosphorescent performance and multicolor reversible photochromism. Owing to their dual photochromism and UOP characteristics, these supramolecular assemblies exhibit great promise for applications in multistage information encryption and time‐resolved photopatterning. This work provides a universal and green supramolecular strategy for constructing multifunctional photo‐responsive materials and opens new avenues for advanced photonic applications.

## Graphical abstract

A concise supramolecular assembly strategy using α‐cyclodextrin and phenylpyridine derivatives affords dual‐functional materials with switchable multicolor photochromism and tunable ultralong organic phosphorescence, offering new platforms for various photonic applications.

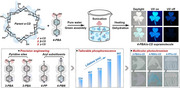

## 1 Introduction

Supramolecular photochromic materials have garnered extensive research attention owing to their unique photo‐active modulatability [1, 2], exhibiting immense potential across diverse advanced fields including optical display [3, 4], information security [5, 6], intelligent sensing [7, 8], and biomedicine [9, 10]. Among various external stimuli, light irradiation stands out with inherent merits including cleanliness, rapid response, remote operability, and high spatiotemporal precision, thus establishing itself as the preferred trigger for modulating supramolecular photochromism [11, 12]. Traditional supramolecular photochromic systems predominantly rely on photo‐induced structural transformations of specific photofunctional molecules, such as the cis‐trans isomerization of azobenzenes [13, 14], ring‐opening/closing of diarylethenes [15, 16], and ring‐closure‐to‐opening isomerization of spiropyrans [17, 18], among others [19, 20]. These systems, however, inherently require ample molecular conformational freedom for rearrangement, a constraint that severely restricts their practical applicability. While efficient photochromism can be achieved in solutions or gels, close molecular packing in the solid state hinders conformational flipping and structural reorganization, compromising photoresponse efficiency and limiting deployment in solid crystals and rigid matrices [21, 22]. In sharp contrast, radical‐dependent photochromism based on photoinduced electron transfer (PET) offers distinctive advantages by overcoming the bottleneck of solid‐state photochromism, reducing reliance on molecular conformational freedom, and maintaining high‐contrast color change, thereby paving a new avenue for fabricating high‐performance solid photochromic materials [23]. For instance, Zhu et al. proposed a universal crystal engineering strategy using hydroxyl‐modified persulfurated arenes, enabling rapid generation and ultra‐stable retention of photoinduced radicals (PIRs) in the solid state via a robust non‐covalent interaction network [24]. Liu and co‐workers, meanwhile, developed a series of solid supramolecular materials with reversible photochromism and tunable fluorescence through host‐guest interactions between viologen derivatives and cucurbituril macrocycles [25, 26, 27]. These studies underscore the pivotal role of PET‐generated PIRs in constructing supramolecular solid‐state photochromic and photo‐modulated fluorescent materials, laying a solid theoretical and experimental foundation for designing novel photo‐responsive supramolecular systems.

In contrast to the prompt emission of conventional fluorescence, room‐temperature ultralong organic phosphorescence (UOP) features a notably longer lifetime and unique persistent afterglow, thereby endowing it with considerable value in advanced photonics and materials science [28, 29]. The marriage of PIRs and RTP has emerged as a burgeoning research frontier, offering unprecedented opportunities for dynamic photo‐responsive luminescent materials [30, 31]. The reversible regulation of phosphorescence via light‐driven generation and quenching of organic radicals enables the construction of dynamic supramolecular RTP systems, as this strategy obviates the need to couple conventional phosphors with photofunctional molecules and avoids the complex design associated with phosphorescence modulation via conformational or absorbance changes [32, 33]. The use of a single molecule with both photoinduced radical generation and phosphorescence emission as the building block further simplifies and upgrades photo‐regulated dynamic supramolecular RTP systems [34, 35]. Notably, relevant advances have been demonstrated by the Liu group, who fabricated flexible films via non‐covalent crosslinking of 4‐cyanophenylpyridinium‐acrylamide copolymers, sulfobutylether‐β‐cyclodextrin, and polyvinyl alcohol to achieve reversible afterglow and photochromism with dual photo‐thermal responsiveness [36]. Yan et al. prepared zero‐dimensional organic‐inorganic halide hybrid glasses doped with 4,4'‐bipyridine via a grinding‐melting‐quenching method, realizing ultralong phosphorescence modulation through a PIR‐induced photochromic switch [37]. The Ma group copolymerized benzothiadiazole‐modified viologen derivatives with acrylamide, preserving viologen photochromism while achieving photo‐responsive RTP emission [38]. Despite these advances, mainstream strategies for such photo‐controlled phosphorescent‐photochromic bifunctional materials still rely on polymerization or multi‐component doping into rigid polymer matrices, which involve cumbersome design and fabrication workflows (Table S1). Consequently, the development of simple, green, and efficient supramolecular photochromic afterglow systems still remains a critical challenge in this field.

**SCHEME 1:**
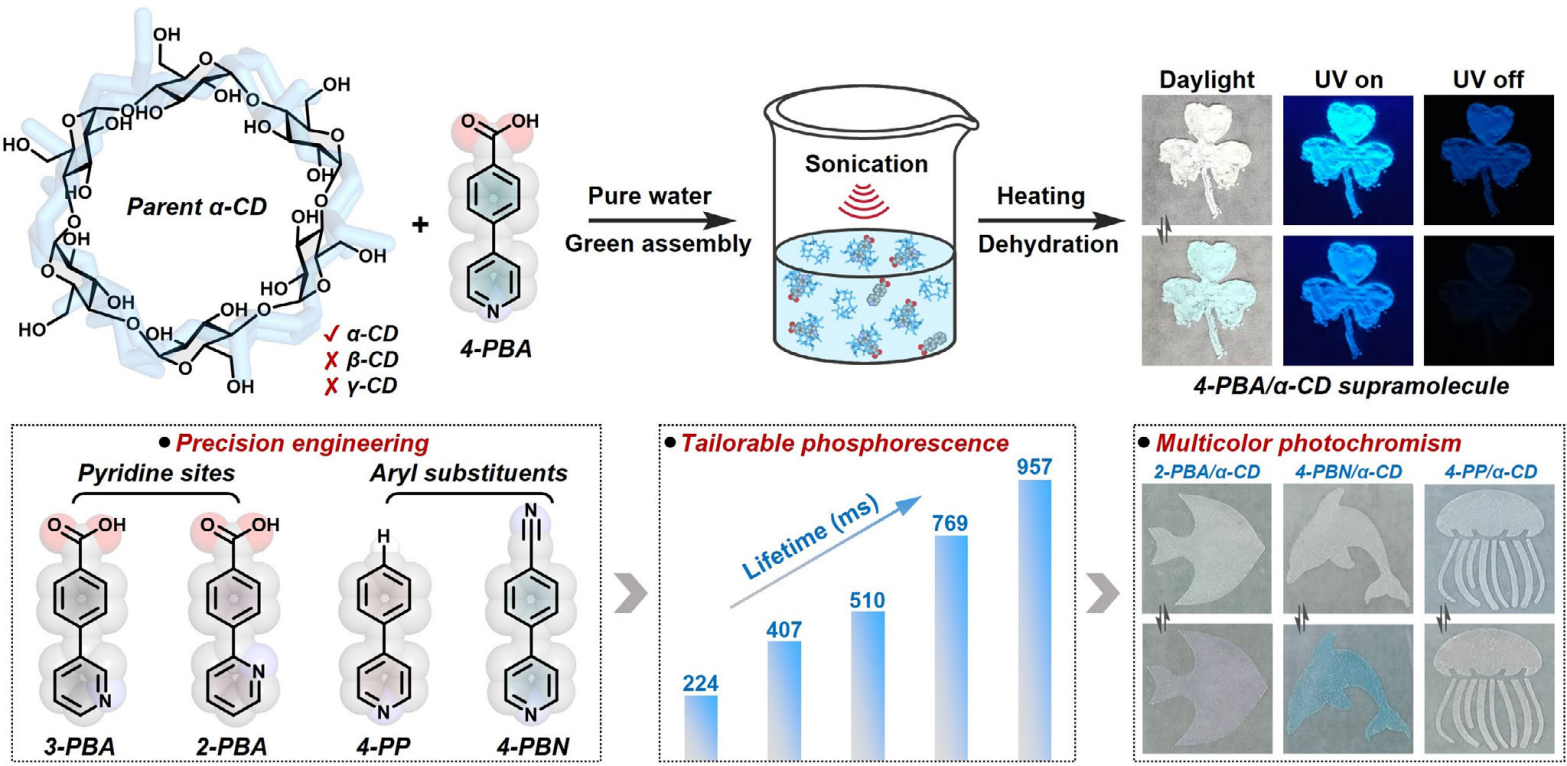
Schematic illustration of the fabrication of 4‐PBA/α‐CD solid supramolecule featuring both efficient ultralong organic phosphorescence (UOP) and reversible photochromism, as well as the precision engineering of guest molecules that afforded a series of supramolecules with tailorable UOP performance and multicolor photochromic responses.

In this context, we reported a concise supramolecular strategy to achieve both efficient UOP and reversible photochromism in a two‐component assembly in the solid state, based on noncovalent interactions between α‐cyclodextrin (α‐CD) and 4‐(pyridin‐4‐yl)benzoic acid (4‐PBA) (Scheme 1). The size‐matched host‐guest inclusion, assisted by hydrogen bonding, provided rigid conformational confinement and an ordered assembly microenvironment that simultaneously suppressed non‐radiative decay to enable a long phosphorescence lifetime up to 0.96 s and promoted reversible PET to generate stable radicals, thereby allowing dynamic and reversible modulation of the afterglow. Through systematic structural, photophysical, and photochromic characterizations, we explored the synergistic mechanism between UOP and photochromism, verified the universality of this strategy with various phenylpyridine derivatives, and further demonstrated its practical application potential in multistage information security and multistate photopatterning. This work is expected to provide a new perspective for the rational design of high‐performance photo‐responsive supramolecular materials with dual photochromic and UOP functionalities.

## 2 Results and Discussion

### 2.1 Structural Characterization of Supramolecule

**FIGURE 1:**
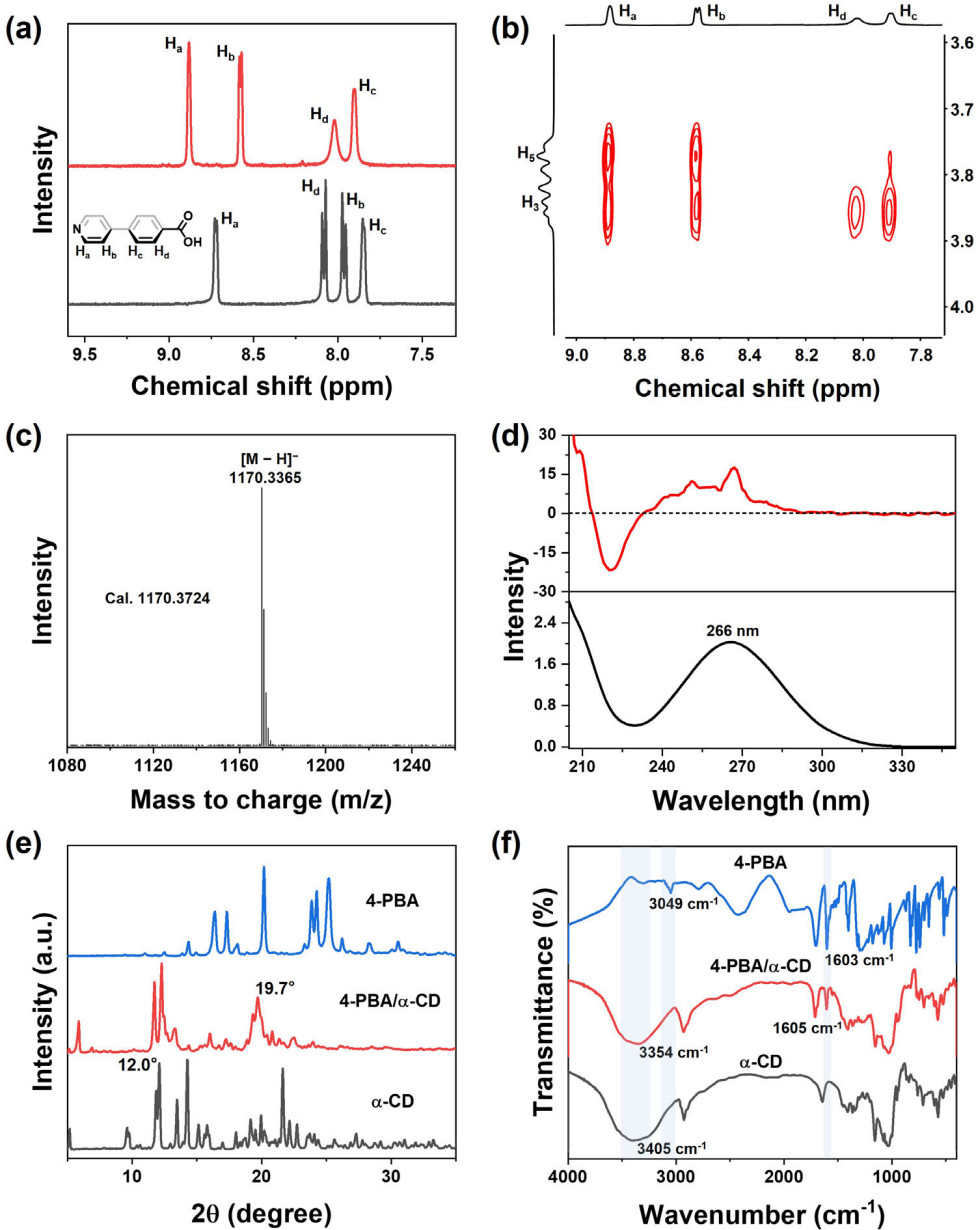
(a) Partial ^1^H NMR (400 MHz, 298 K) spectra of 4‐PBA/α‐CD in D_2_O and free 4‐PBA in DMSO‐d_6_. (b) 2D NMR (ROESY, 400 MHz, 298 K) of 4‐PBA/α‐CD in D_2_O. (c) High‐resolution ESI‐MS mass spectrum of 4‐PBA/α‐CD. The peak at m/z 1170.3365 corresponded to [M－H]^−^. m/z calcd for C_48_H_68_NO_32_^−^ 1170.3724. (d) Circular dichroism and UV‐vis absorption spectra of 4‐PBA/α‐CD. (e) Powder X‐ray diffraction patterns of 4‐PBA, α‐CD, and 4‐PBA/α‐CD, respectively. (f) FT‐IR spectra of 4‐PBA, α‐CD, and 4‐PBA/α‐CD, respectively.

To investigate the noncovalent interaction between α‐cyclodextrin (α‐CD) and 4‐(pyridin‐4‐yl)benzoic acid (4‐PBA), ^1^H NMR experiments were first performed. NMR spectroscopy revealed that all aromatic protons of 4‐PBA shifted downfield after complexation, with the pyridine ring protons (H_a,b_) exhibited a more pronounced upfield shift, indicating their insertion into the hydrophobic cavity of α‐CD, while the benzoic acid protons (H_c_) showed a smaller shift (Figure 1a and Figure S1). Particularly, 2D rotating frame overhauser effect spectroscopy (ROESY) provided definitive insights into the precise orientation of 4‐PBA within the α‐CD cavity (Figure 1b), which showed that the protons of the pyridine ring (H_a,b_) exhibited strong nuclear overhauser effect (NOE) cross‐peaks with both H_3_ and H_5_ of α‐CD. This indicated that the pyridine moiety was deeply inserted into the cavity, in close proximity to both the wider rim (H_3_) and the narrower inner core (H_5_). In contrast, the benzene ring protons (H_c,d_) only correlated with H_3_, confirming that the benzene ring was partially embedded near the wider rim, while the carboxyl group extended outside the cavity, facilitating hydrogen bond formation. The ESI‐TOF mass spectrum furnished direct molecular‐level evidence for the formation of a well‐defined 1:1 host‐guest inclusion complex between 4‐PBA and α‐CD. A prominent peak at m/z = 1170.3365 was observed (Figure 1c), corresponding to the deprotonated ion [M−H]^−^ of the 4‐PBA/α‐CD complex. The theoretical m/z value for this species was calculated as 1170.3724, confirming the identity of the 1:1 inclusion complex. Analysis of the phase solubility profile indicated that the apparent stability constant for the 4‐PBA/α‐CD inclusion complex was about 3.01 × 10^3^ M^−1^ (Figure S2). The UV–vis spectrum of 4‐PBA/α‐CD showed characteristic absorption bands at 230–330 nm, corresponding to the π–π* transitions of the aromatic backbone of 4‐PBA (Figure 1d, bottom). Meanwhile, the circular dichroism spectrum exhibited a distinct induced negative Cotton effect at 220 nm and a positive Cotton effect from 235 to 292 nm (Figure 1d, top), which were absent in the spectrum of free 4‐PBA (Figure S3), an achiral molecule. These induced circular dichroism signals arose from the chiral confinement of the achiral 4‐PBA guest within the chiral cavity of α‐CD, providing direct evidence for the formation of a well‐defined host‐guest inclusion complex with a fixed molecular orientation and conformation.

The powder X‐ray diffraction (XRD) patterns provided compelling evidence for the formation of a new ordered supramolecular crystalline phase between 4‑PBA and α‑CD (Figure 1e). The characteristic diffraction peaks of neat 4‑PBA and α‑CD were largely absent in the 4‑PBA/α‑CD complex, indicating the destruction of their individual crystal structures and the absence of simple physical mixing. Notably, a new distinct diffraction peak appeared at 2θ = 19.7° in the complex, which was attributed to the formation of the host‐guest inclusion complex with a long‑range ordered arrangement. Furthermore, the disappearance of the characteristic peak of α‑CD at 2θ = 12.0° confirmed a significant change in the crystal packing of α‑CD from a cage‑type to a channel‑type structure. Moreover, as the cyclodextrin cavity size increased, the diffraction peaks of the inclusion complexes gradually broadened, and their crystalline order decreased (Figure S4). Notably, the crystalline peaks of the 4‐PBA/γ‐CD inclusion complex became almost fully diffuse, which directly reflected the regulatory effect of cyclodextrin cavity size on the molecular packing and crystallization behavior of the supramolecules. This channel‐type assembly was further supported by Fourier transform infrared (FT‐IR) spectroscopy (Figure 1f). The characteristic Ar–H stretching peak of 4‐PBA at 3049 cm^−1^ disappeared upon complexation, accompanied by a pronounced reduction in the intensity of the aromatic C═C stretching band at 1603 cm^−1^. These spectral changes arose from the greatly restricted vibrational freedom and diminished dipole moment change of the aromatic moiety after being embedded within the hydrophobic cavity of α‐CD. Concurrently, the O–H stretching band of α‐CD showed a distinct red shift from 3405 to 3354 cm^−1^, verifying the formation of intermolecular hydrogen bonds between the carboxyl groups of 4‐PBA and the hydroxyl groups of α‐CD at the channel periphery.

### 2.2 Ultralong Organic Phosphorescence (UOP)

**FIGURE 2:**
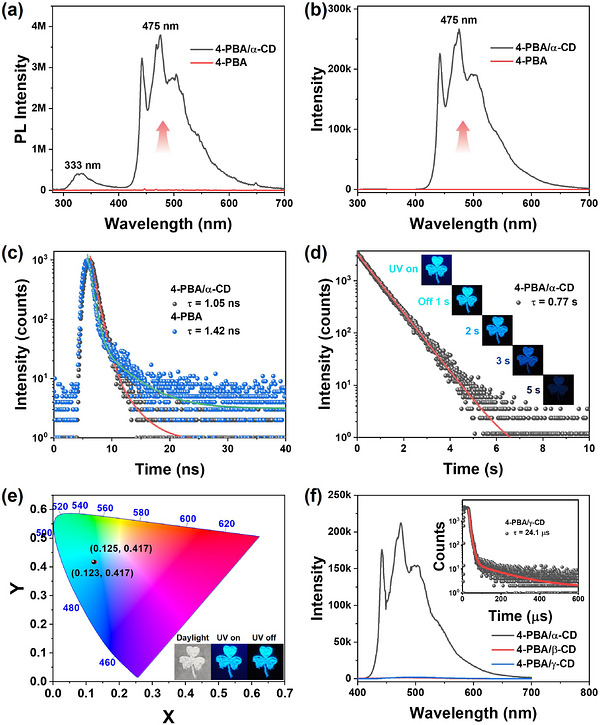
(a) The prompt photoluminescence spectra of 4‐PBA and 4‐PBA/α‐CD. (b) The gated emission spectra (delay 1 ms) of 4‐PBA and 4‐PBA/α‐CD (λ_ex_ = 270 nm). (c) Time‐resolved photoluminescence decay spectra of 4‐PBA and 4‐PBA/α‐CD at 400 nm and 333 nm at 298 K, respectively. (d) Time‐resolved photoluminescence decay spectrum of 4‐PBA/α‐CD at 475 nm at 298 K. Inset: Photographs of the solid sample during 254 nm UV illumination and immediately after switching off the light. (e) The CIE 1976 chromaticity diagram recorded the color variations of the 4‐PBA/α‐CD system upon UV exposure and cessation, and the inset provided the corresponding photographic images. (f) The gated emission spectra (delay 1 ms) of the 4‐PBA/α‐CD, 4‐PBA/β‐CD, and 4‐PBA/γ‐CD, respectively, where the inset was the corresponding time‐resolved photoluminescence decay spectrum of 4‐PBA/γ‐CD at 298 K.

To eliminate potential interference from residual impurities on phosphorescence generation, high‐performance liquid chromatography (HPLC) analysis was conducted on the guest molecule 4‐PBA (Figure S5), which confirmed the high purity of 4‐PBA, ensuring reliable interpretation of its photophysical properties in subsequent studies. The photoluminescent properties of the pristine solid 4‐PBA/α‐CD supramolecule were investigated in detail. Pure solid 4‐PBA displayed only an extremely weak prompt fluorescence emission band around 400 nm with a nanosecond‐scale lifetime of 1.42 ns, whereas the solid‐state PL spectrum of 4‐PBA/α‐CD, in sharp contrast, exhibited two well‐resolved emission bands centered on 333 and 475 nm, respectively (Figure 2a,c and Figure S6). In the time‐gated spectrum recorded with a 1 ms time delay, the short‐wavelength prompt emission disappeared completely, with only the long‐wavelength emission remaining (Figure 2b), which confirmed the long‐lived excited‐state nature of the low‐energy emission. Corresponding to these bands, the photoluminescent emission of the sample was nearly indistinguishable from its afterglow emission, and even 5 s following the cessation of UV irradiation, the azure‐blue afterglow remained visible (Figure 2d, inset). The CIE 1976 chromaticity diagram further depicted the photoluminescent and afterglow color distribution as the chromaticity coordinates shifted from (0.123, 0.417) to (0.125, 0.417) (Figure 2e). Time‐resolved PL decay measurements further showed that the long‐wavelength emission exhibited a lifetime of 0.77 s in the second timescale (Figure 2d), typical of triplet excited‐state emission, while the short‐wavelength component displayed a nanosecond‐scale lifetime of 1.05 ns (Figure 2c), characteristic of prompt fluorescence. It was likely that the reduction in fluorescence lifetime arose because numerous singlet excitons underwent rapid conversion to the triplet state, thus accelerating the decay of singlet‐state populations. Notably, upon exposure to an aqueous environment, the long‐wavelength emission intensity was drastically quenched to some degree, a typical feature of triplet‐involved emission caused by severe water quenching (Figure S7). Nevertheless, this emission band retained a relatively long lifetime of 40.7 ms (Figure S8). Collectively, these results confirmed that the long‐wavelength emission band arose from room‐temperature phosphorescence, while the short‐wavelength band was assigned to conventional prompt fluorescence from the singlet excited state. Particularly, this supramolecular system achieved a high phosphorescence quantum yield of 22.0% (Figure S9), indicating highly efficient triplet harvesting and radiative recombination.

The exceptional ultralong phosphorescent afterglow performance could be attributed to the synergistic hydrogen bonding and favorable host‐guest size matching between the hydrophobic cavity of α‐CD and the aromatic 4‐PBA, which imposed stringent spatial restriction on the encapsulated 4‐PBA molecules, effectively suppressing intramolecular vibration and rotation. Such rigid conformational confinement significantly attenuated the non‐radiative deactivation pathway that would otherwise dissipate the excitation energy of triplet excitons rapidly. Furthermore, the 4‐PBA/α‐CD complex self‐assembled into an ordered channel‐type structure, where the hydrophobic cavities aligned linearly to form a protective microenvironment (Figure 1e,f). This ordered assembly afforded effective isolation against atmospheric oxygen and moisture, which were prominent triplet exciton quenchers, thereby extending the lifetime of triplet excited states. In sharp contrast, the absence of rigid confinement led to intense molecular motions for neat solid 4‐PBA accompanied by severe quenching effects, collectively accelerated non‐radiative decay of triplet excitons and completely quenched phosphorescence emission (Figure 2b and Figure S10), resulting in the absence of observable UOP with persistent afterglow. For the 4‐PBA/β‐CD and 4‐PBA/γ‐CD supramolecules, the oversized cavities of β‐CD and γ‐CD led to loss of encapsulation of 4‐PBA guest molecules, failing to provide sufficient conformational restriction to suppress non‐radiative relaxation. Additionally, the lack of ordered packing in 4‐PBA/β‐CD and 4‐PBA/γ‐CD prevented effective isolation of triplet excitons from oxygen quenching, and the irregular intermolecular arrangement hindered efficient triplet stabilization (Figure S4). Consequently, neither 4‐PBA/β‐CD nor 4‐PBA/γ‐CD exhibited significant photoluminescence or detectable UOP behavior (Figure 2f and Figure S11), while their lifetimes were determined to be 3.03 µs and 24.1 µs on the microsecond time scale under identical conditions, respectively (Figure 2f, inset and Figure S12). Moreover, 4‐PBA/α‐CD still exhibited stronger emission intensity in aqueous media than its β‐ and γ‐CD counterparts (Figure S7), further demonstrating that α‐CD effectively protected 4‐PBA from non‐radiative deactivation induced by water molecules. This comparison further highlighted that only a size‐matched and ordered structural microenvironment from α‐CD can enable efficient triplet harvesting and UOP emission.

### 2.3 Photochromic Properties

**FIGURE 3:**
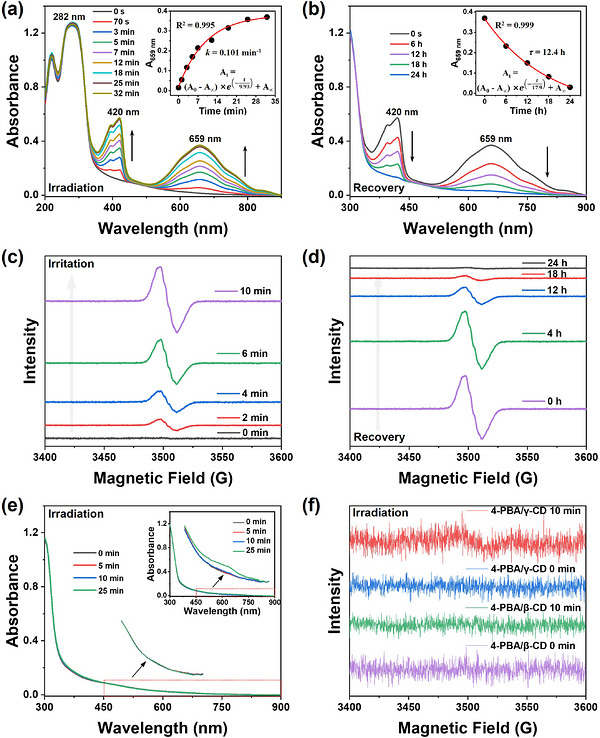
(a) UV–vis absorption spectra of 4‐PBA/α‐CD upon continuous UV irradiation (254 nm, 3 W). Inset: Corresponding time‐dependent spectral evolution profile at 659 nm. (b) UV‐vis absorption spectra of the post‐irradiated 4‑PBA/α‑CD during the recovery process under ambient conditions. Inset: Corresponding time‐dependent spectral evolution profile at 659 nm. (c) Electron paramagnetic resonance (EPR) spectra of 4‐PBA/α‐CD upon continuous UV irradiation. (d) EPR spectra of the post‐irradiated 4‑PBA/α‑CD during the recovery process under ambient conditions. (e) UV‐vis absorption spectra of 4‐PBA/β‐CD and 4‐PBA/γ‐CD (inset) upon continuous UV irradiation. (f) EPR spectra of 4‐PBA/β‐CD and 4‐PBA/γ‐CD before (0 min) and after (10 min) UV irradiation.

**FIGURE 4:**
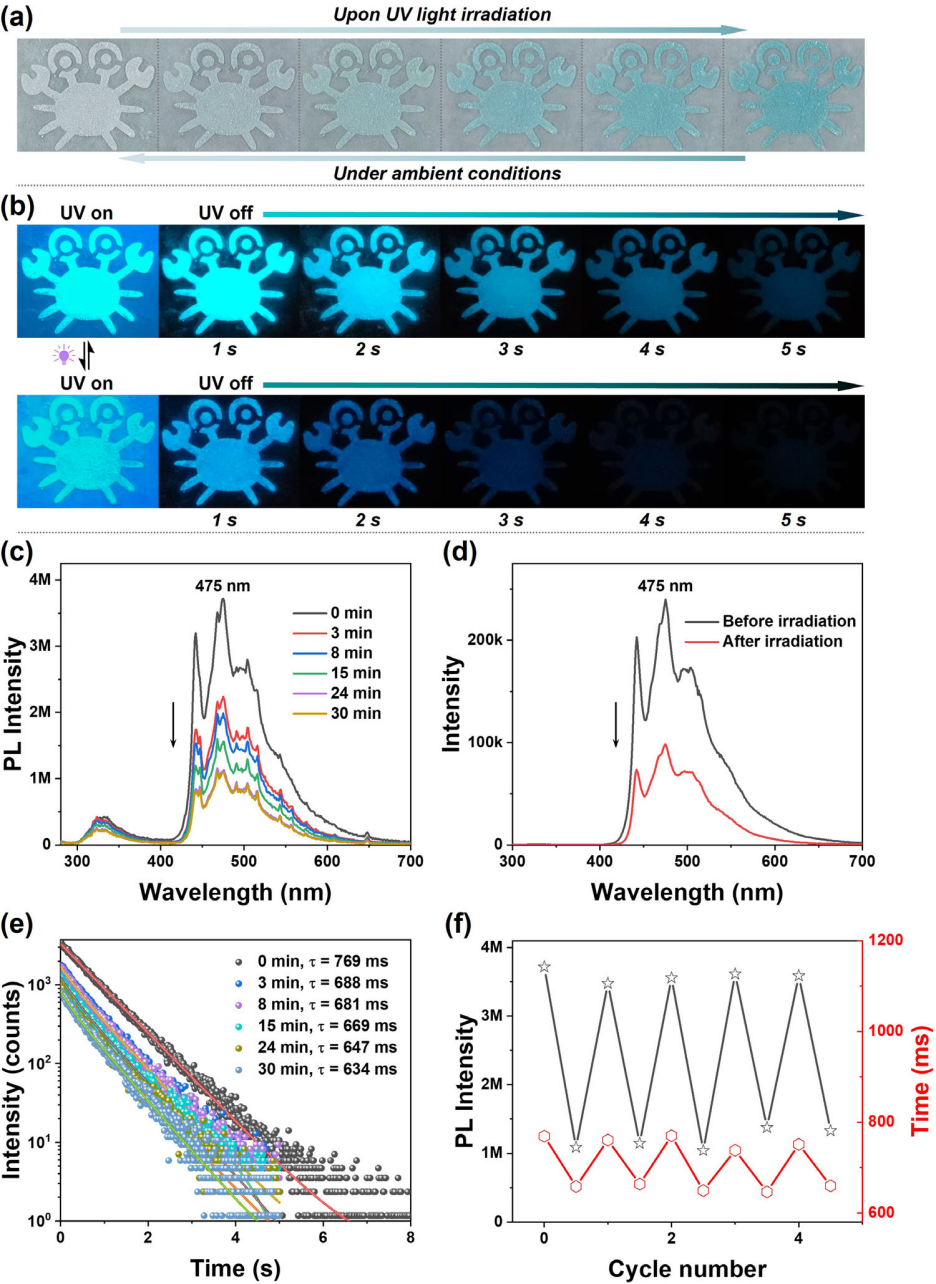
(a) Photographs of the photochromic color changes of 4‐PBA/α‐CD under ambient conditions. Unless otherwise stated, all photochromic investigations in this study were carried out under an air atmosphere. (b) Photographs illustrating the distinct afterglow characteristics of 4‑PBA/α‑CD before and after photochromism. (c) The prompt photoluminescence spectra of 4‐PBA/α‐CD upon continuous UV irradiation. (d) The gated emission spectra (delay 1 ms) of 4‐PBA/α‐CD before and after UV irradiation. (e) Time‐resolved photoluminescence decay spectra of 4‐PBA/α‐CD at 475 nm at 298 K upon continuous UV irradiation. (f) Changes in photoluminescence intensity and lifetime of 4‑PBA/α‑CD at 475 nm under alternating UV irradiation and recovery conditions.

Interestingly, in addition to UOP behavior, 4‐PBA/α‐CD presented distinctive photo‐responsive behavior: under continuous UV irradiation, the solid 4‐PBA/α‐CD displayed a striking visual color transition from pristine white to cyan blue under indoor light because it absorbed its complementary light (Figure 4a). Thus, UV–vis spectroscopy was utilized to investigate the solid‐state photochromic process of supramolecules. As depicted in Figure 3a, the as‐prepared 4‐PBA/α‐CD presented an absorption band centered at 282 nm attributed to π–π* transitions prior to light exposure. After UV irradiation, two new long‐wavelength absorption bands gradually emerged at 420 nm and 659 nm, with the absorption intensity reaching its saturation in 25 min. These new absorption features in the visible region corresponded well with the color appearance of UV‐saturated 4‐PBA/α‐CD, implying the generation of new chemical species. To further quantify such a rapid photochromic process, the apparent rate constant (k) was determined to be 0.101 min^−1^ by employing the kinetic model ln[(A_0_−A_∞_)/(A_t_−A_∞_)] = kt, based on the time‐dependent absorbance monitored at 659 nm (Figure 3a, inset). To identify the origin of the photochromic response, PL excitation spectrum, FT‐IR, and powder XRD patterns of the fully photoactivated sample were compared with those of the pristine sample (Figures S13 and S14), where all spectra were nearly identical, ruling out molecular isomerization, chemical bond formation/cleavage, or crystal phase transitions. Combined with the structural characteristics of 4‐PBA/α‐CD, it was speculated that the newly emerged absorption bands most likely originated from photoinduced radicals generated via a photoinduced electron transfer (PET) process, which required extremely low molecular conformational freedom and even no obvious conformational changes [23, 24].

Subsequently, electron paramagnetic resonance (EPR) measurements were performed to directly verify the formation of radicals. As expected, no obvious signal was detected in the EPR spectrum of the unirradiated sample, whereas a gradually intensifying symmetrical single‐line resonance signal with a g‐value of 2.007 appeared upon continuous UV photoactivation from 2 to 10 min (Figure 3c). This g‐value was basically consistent with that of a free electron (i.e., 2.0023), confirming the generation and accumulation of photoinduced radicals. The observed EPR signal could be attributed to the formation of organic free radicals, which was presumed to stem from photo‐induced electron transfer between the oxygen atoms of the host α‐CD and the pyridine segment in the guest structures [39, 40]. Particularly, the enhancement of the EPR signal occurred synchronously with the gradual deepening of color during the photochromic process, verifying the direct correlation between radical formation and color change. To eliminate the possibility of photooxidation by ambient oxygen, irradiation of 4‐PBA/α‐CD was also carried out in a glove box under anhydrous and deoxygenated conditions, and the same color change and distinct radical signal were observed immediately upon light exposure (Figure S15), clearly ruling out the involvement of environmental oxygen in the photochromic process. Notably, after 24 h of dark storage, the light‐induced coloration of 4‐PBA/α‐CD slowly reverted to its original white state, and this coloring‐fading cycle could be repeated multiple times without obvious color loss (Figure S16). This exceptional stability strongly implied that the formation of long‐lived photogenerated radical species was the key driver behind the observed photochromism. After decolorization, all characteristic absorption bands at wavelengths above 400 nm completely disappeared, and the spectra of the sample reverted to their initial states (Figure 3b). The lifetime of the photogenerated persistent radicals was further evaluated from the absorbance decay profile recorded at 659 nm. By fitting the kinetic data to the model ln[(A_0_−A_∞_)/(A_t_−A_∞_)] = k't, the decay rate constant k' was calculated to be 0.056 h^−1^, corresponding to a half‐life τ of 12.4 h, as derived from the relationship τ = ln2/k' (Figure 3b, inset). Furthermore, consistent with the fading behavior, EPR spectroscopic analysis of 4‐PBA/α‐CD showed that the intensity of the characteristic radical signal gradually decreased until complete disappearance during the decolorization process (Figure 3d). These experimental results collectively confirmed that the sample exhibited excellent photochromic reversibility in terms of reversible radical generation and quenching. Notably, the FT‐IR and powder XRD patterns of the recovery sample after irradiation were consistent with their initial state before irradiation (Figure S14), which further ruled out structural transformation, isomerization, or phase change as the intrinsic cause of the reversible photochromic process.

However, neat solid 4‐PBA showed no reversible photochromic response under irradiation (Figure S17), which was probably attributed to its dense and disordered molecular packing that caused uncontrollable electron transfer and failed to stabilize photogenerated radicals. For 4‐PBA/β‐CD and 4‐PBA/γ‐CD, their much larger cavities resulted in loose encapsulation and excessive molecular separation. Such structural features impeded efficient and reversible intermolecular electron transfer, failing to generate stable photogenerated radicals. As shown in Figure 3e, the UV–vis absorption spectra of both supramolecules remained nearly unchanged throughout 25 min of irradiation, with no discernible increase in absorbance in the visible region, indicating the absence of photochromic behavior. Furthermore, EPR measurements revealed no detectable radical signals before or after 10 min of light exposure, confirming the lack of persistent radical generation (Figure 3f and Figure S18). Neither 4‐PBA/β‐CD nor 4‐PBA/γ‐CD exhibited obvious photo‐responsive behaviors under identical experimental conditions (Figure S19). In another comparative experiment, 4‐PBA in PVA matrices showed moderate UOP performance with a 302 ms lifetime but no obvious photochromic response under light irradiation, which suggested that in addition to the abundant hydroxyl groups, the unique conical structure of α‐CD was essential to the photochromic behavior (Figure S20). Therefore, similar to the UOP performance, such distinct solid‑state photochromism was exclusive to 4‐PBA/α‐CD, which was closely related to α‐CD structural support. For one thing, the size‐matched cavity enabled tight 1:1 inclusion of 4‐PBA and ordered 1D channel packing, providing moderate and uniform intermolecular distances for a controllable, reversible PET process. For another, the 1D channel confinement shielded radical species from quenchers, enabling radical accumulation and the formation of characteristic visible absorption responsible for color change. Besides, the hydrogen‐bonding network at the α‐CD cavity rim further stabilized photogenerated radicals, thus facilitating reversible coloration and bleaching.

### 2.4 Reversible Photochromic UOP

Given the shared dependence of UOP and photochromism on the α‐CD microenvironment and triplet exciton dynamics, we hypothesized a synergistic interplay between these two phenomena. Specifically, the reversible photochromism, driven by the generation and quenching of radicaloid species, was anticipated to act as an effective optical switch to dynamically modulate the UOP performance via a photo‐controllable regulatory manner. Experimental observations confirmed this synergistic regulation. With increasing duration of UV irradiation, the intensities of both the prompt fluorescence at 333 nm and the RTP emission at 475 nm declined significantly (Figure 4c,d). This attenuation was accompanied by a visible color transition of the sample from pale white to a deep cyan (Figure 4a), consistent with the progression of photochromism. Prior to photochromic conversion, a green afterglow was clearly visible to the naked eye for 5 s after UV cessation. After 30 min of continuous irradiation, the afterglow duration was shortened to approximately 3 s (Figure 4b). Time‐resolved PL decay measurements further revealed a reduction in the RTP lifetime from an initial value of 769 ms to 634 ms (Figure 4e). It was noted that the phosphorescence lifetime underwent attenuation to a certain extent following the photochromic response, which was probably because such photochromic phenomena existed only on the surface of the bulk solid supramolecule [41]. Notably, when the colored sample reverted to its original white appearance following dark storage, the PL spectrum, RTP intensity, and RTP lifetime fully recovered (Figure 4f), with the restoration of RTP characteristics being particularly distinct. These results demonstrated that the reversible photochromism of 4‐PBA/α‐CD played a critical role in regulating its photoluminescent behavior, especially its RTP characteristics. The reversible RTP switching accompanied the coloration‐fading cycles and remained stable for at least five consecutive cycles (Figure 4f and Figures S21 and S22), highlighting the feasibility of modulating solid‐state supramolecular afterglow through a photochromic process.

This modulation effect could be attributed to two coupled pathways, both governed by the competition and interplay between triplet excitons and photogenerated radicals [42, 43]. On the one hand, upon UV excitation, singlet excitons were generated and efficiently converted to long‐lived triplet excitons via intersystem crossing. This process was promoted by the rigid conformational confinement imposed by the α‐CD matrix. The short‐lived singlet state was insufficiently long‐lived to enable efficient PET process. Consequently, the photochromic radicals were predominantly derived from long‐lived triplet excitons rather than singlet excitons [44]. As a result, triplet excitons underwent two competitive relaxation pathways: radiative relaxation to the ground state, which yielded ultralong RTP and persistent afterglow; and reversible PET process to generate stable radicaloids and trigger photochromism. The observation of a considerably more pronounced decrease in RTP intensity compared to fluorescence intensity during irradiation indicated that triplet excitons were preferentially consumed for radical formation (Figure 4c), leading to a reduction in UOP emission. On the other hand, the observed emission attenuation was further ascribed to non‐radiative energy transfer from triplet excited states to the increasing population of non‐emissive photogenerated radicals. A distinct spectral overlap between the ultralong RTP emission band and the absorption region at 400–700 nm indicated the occurrence of self‐absorption by the photogenerated radical species (Figures 3a,b and 4c,d). The gradual increase in absorption intensity at 659 nm, accompanied by the continuous decline of photoluminescence intensity with prolonged irradiation, confirmed that the enhanced self‐absorption effect contributed to the decay of RTP intensity and lifetime upon photochromic coloration. Collectively, these results verified that radical generation and the modulated light absorption upon photoirradiation were key factors enabling photo‐responsive reversible RTP.

### 2.5 Universal Phenylpyridine Supramolecules for Photochromic UOP

To verify the universality of our supramolecular strategy for constructing photochromic UOP materials, we extended our investigation to additional phenylpyridine derivatives, namely 4‐phenylpyridine (4‐PP), 4‐(pyridin‐4‐yl)benzonitrile (4‐PBN), 4‐(pyridin‐3‐yl)benzoic acid (3‐PBA), and 4‐(pyridin‐2‐yl)benzoic acid (2‐PBA). Similar with 4‐PBA, it could be observed that all four guest molecules displayed a single prompt fluorescence emission band without significant delayed emission (Figures S23–S26). The photophysical properties of 4‐PP and 4‐PBN upon complexation with α‐, β‐, and γ‐CDs were systematically investigated to reveal the synergistic effects of cyclodextrin cavity size and substituent electronic structure on RTP behavior. α‐CD efficiently induced distinct RTP for 4‐PP and 4‐PBN guest molecules, while β‐ and γ‐CD complexes only showed dominant fluorescence with negligible phosphorescent emission (Figures S27 and S28), as the compact α‐CD cavity rigidified the guest molecules, suppressed non‐radiative decay pathways, and stabilized the triplet excited state, whereas the larger cavities of β‐ and γ‐CD allowed enhanced molecular motion that favored non‐radiative deactivation. The phosphorescence lifetimes of these supramolecules ranked as 4‐PP/α‐CD (τ = 957 ms) > 4‐PBA/α‐CD (τ = 769 ms) > 4‐PBN/α‐CD (τ = 224 ms) (Figures S9, S29 and S30), with corresponding phosphorescence quantum yields of 53.1%, 22.0%, and 9.3%, respectively, which correlated well with the electronic nature of the para‐substituent.

**FIGURE 5:**
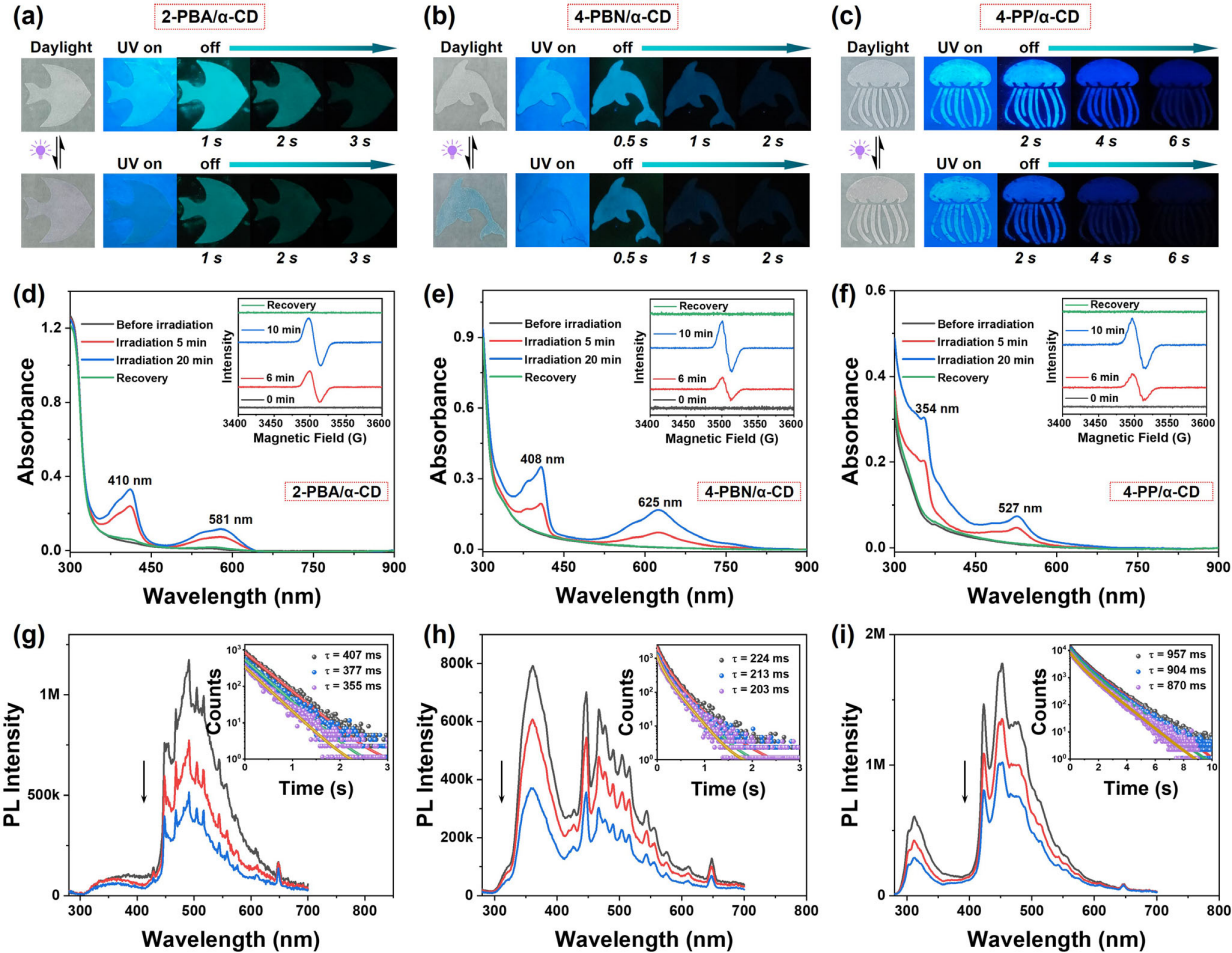
Photographs showing the different afterglow behaviors of (a) 2‑PBA/α‑CD, (b) 4‐PBN/α‐CD, and (c) 4‐PP/α‐CD prior to and following photochromism. UV‐vis spectra of (d) 2‑PBA/α‑CD, (e) 4‑PBN/α‑CD, and (f) 4‑PP/α‑CD under continuous UV irradiation, with the corresponding EPR profiles during ambient recovery shown in the insets. The prompt photoluminescence spectra of (g) 2‑PBA/α‑CD, (h) 4‐PBN/α‐CD, and (i) 4‐PP/α‐CD under continuous UV irradiation, where the insets present the corresponding time‐resolved photoluminescence decay profiles at 298 K.

Moreover, the photoluminescent properties of positional isomers of phenylpyridine carboxylic acids (2‐PBA, 3‐PBA) upon complexation with α‐, β‐, and γ‐CDs were also systematically characterized. Assemblies with β‐ and γ‐CDs showed extremely faint phosphorescence, whereas only α‐CD effectively induced phosphorescence for these guests (Figures S31 and S32). In steady‐state PL spectra, 4‐PBA/α‐CD exhibited intense and structured emission bands from 400 nm to 700 nm, while 2‐PBA/α‐CD showed moderate emission, and 3‐PBA/α‐CD displayed only weak features. Time‐gated PL spectra revealed that phosphorescence intensity followed the order 4‐PBA/α‐CD (τ = 769 ms) > 3‐PBA/α‐CD (τ = 510 ms) > 2‐PBA/α‐CD (τ = 407 ms) (Figure S33). This position‐dependent RTP probably arose from distinct host‐guest spatial matching and conformational rigidity within the α‐CD cavity. Para‐substituted 4‐PBA optimally fitted the compact α‐CD cavity, forming tight inclusion complexes that suppressed non‐radiative decay and stabilized triplet excited states, leading to strong RTP, while ortho‐substituted 3‐PBA and meta‐substituted 2‐PBA experienced reduced cavity compatibility to form sufficiently rigid assemblies. These results highlighted the critical role of substituent position in dictating supramolecular photophysics, where precise spatial matching within the α‐CD cavity enabled efficient triplet stabilization and RTP. The afterglow behaviors of 4‐PP/α‐CD, 4‐PBN/α‐CD, 3‐PBA/α‐CD, and 2‐PBA/α‐CD could be seen in Figure 5a–c and Figure S34. Collectively, these results demonstrated that the rigid microenvironment provided by α‐CD, combined with substituent electronic effects and positional effects, synergistically controlled triplet‐state dynamics and RTP performance. This dual‐control strategy offered a rational approach for the design of high‐performance organic RTP materials through precise supramolecular engineering.

Consistent with their robust UOP performance, the α‐CD complexes of 2‐PBA, 4‐PBN, and 4‐PP also exhibited distinct photochromic behavior upon light irradiation, as confirmed by UV‐vis and EPR spectroscopy (Figure 5d–f). Upon continuous irradiation, new absorption bands emerged in the visible region for 2‐PBA/α‐CD (410 and 581 nm), 4‐PBN/α‐CD (408 and 625 nm), and 4‐PP/α‐CD (354 and 527 nm), accompanied by a visible color change from their initial off‐white appearance to pale purple, sky blue, and light pink, respectively. These new bands were attributed to the formation of stable photogenerated radicaloid species, which was directly verified by EPR measurements: pronounced radical signals appeared after 6 min of irradiation and intensified with prolonged exposure (10 min), and these EPR signals were fully reversible upon dark storage for 24 h, in perfect agreement with the complete recovery of the visible absorption bands observed in UV‐vis spectra, confirming the reversible nature of radical formation and quenching. In an anhydrous and deoxygenated glove box, irradiation of the 2‐PBA /α‐CD, 4‐PBN/α‐CD, and 4‐PP/α‐CD was conducted to rule out oxygen‐mediated photooxidation, yielding identical color changes and distinct radical signals immediately upon light exposure (Figure S35).

Furthermore, FT‐IR and XRD measurements revealed no discernible changes for 2‐PBA/α‐CD, 4‐PBN/α‐CD, and 4‐PP/α‐CD before irradiation, after light exposure, and following complete recovery in the dark (Figures S36 and S37), confirming that the photochromic process occurred without irreversible structural degradation and remained fully reversible at the molecular level. In sharp contrast, solid 3‐PBA, 2‐PBA, 4‐PBN, 4‐PP, and 3‐PBA/α‐CD showed no discernible photochromic response under identical irradiation conditions (Figures S34 and S38). The UV–vis absorption spectra of 3‐PBA/α‐CD remained nearly unchanged across 300–900 nm, and no detectable EPR radical signals were observed before or after light exposure, even after prolonged irradiation (Figure S39). The absence of photochromic behavior of 3‐PBA/α‐CD probably originated from the intrinsic molecular structure of 3‐PBA, which disfavored the generation of photogenerated radicals even after assembly with α‐CD. Notably, the visible‐region absorbance increments for 2‐PBA/α‐CD, 4‐PBN/α‐CD, and 4‐PP/α‐CD were all lower than those of 4‐PBA/α‐CD under identical irradiation conditions. This result indicated that 4‐PBA/α‐CD generated a higher concentration of stable photogenerated radicals, consistent with its superior photochromic response and stronger UOP relative to the other analogues. In line with 4‐PBA/α‐CD, the 2‐PBA/α‐CD, 4‐PBN/α‐CD, and 4‐PP/α‐CD also enabled reversible modulation of their UOP performance via photochromism, as evidenced by the gradual quenching of phosphorescence intensity upon light irradiation and its partial recovery upon dark storage (Figure 5g–i and Figure S40). The phosphorescence lifetimes of these supramolecules also exhibited a certain degree of reduction after prolonged irradiation. This robust photomodulation behavior observed across multiple phenylpyridine derivatives with varied substituents and substitution positions fully validated the versatility of our supramolecular strategy for constructing photo‐responsive UOP materials, where the rigid microenvironment from multivalent noncovalent interactions synergistically enabled both efficient triplet stabilization and reversible radical‐based photochromism.

### 2.6 Practical Applications of Photochromic UOP

**FIGURE 6:**
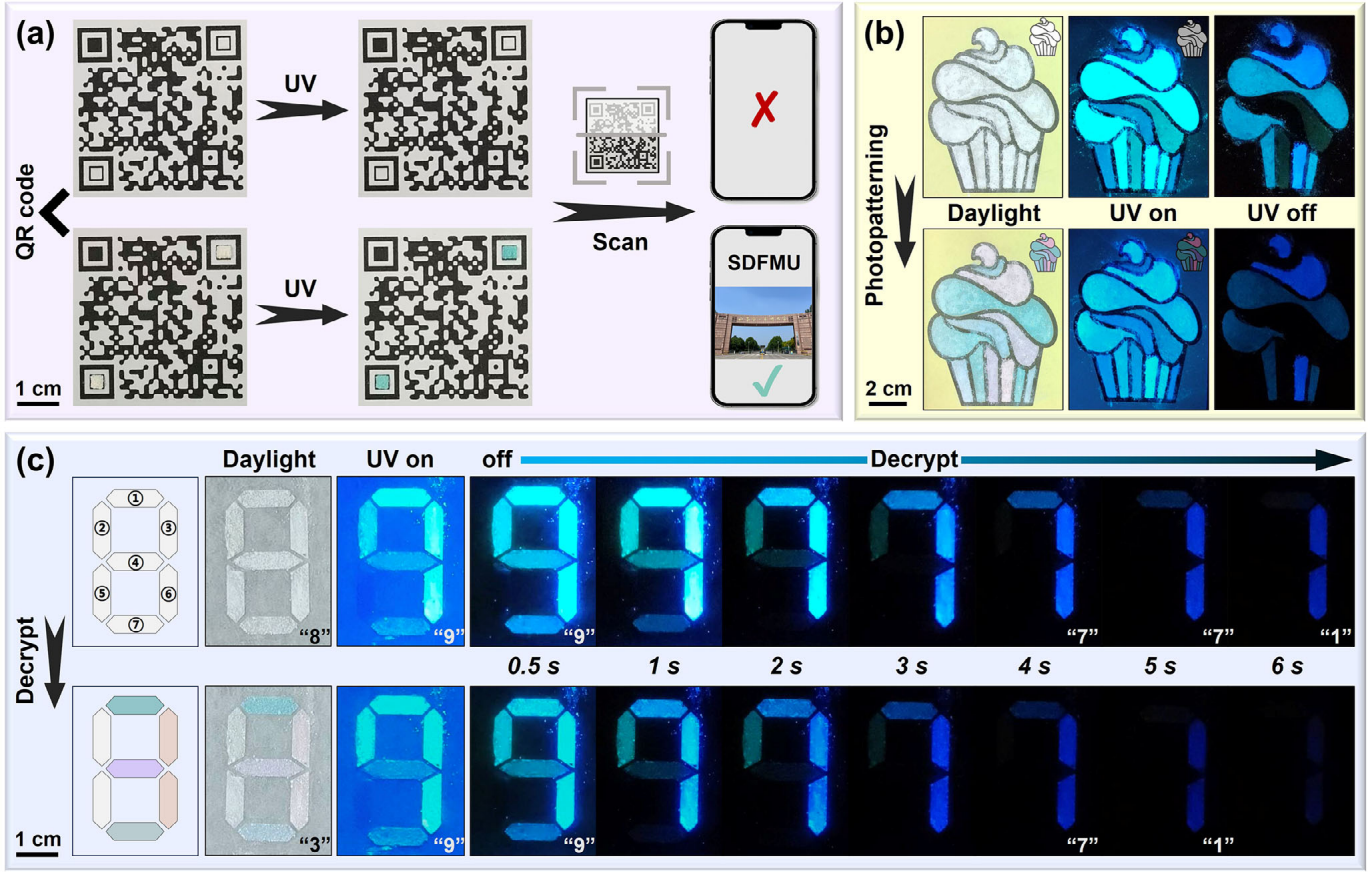
(a) Photochromic QR code anti‐counterfeiting based on 4‐PBA/α‐CD solid supramolecule. (b) Time‐resolved photopatterning of cupcake patterns (daylight, UV on, and 2s after UV off) using different phenylpyridine/α‐CD solid supramolecules with photochromic and phosphorescent responses. (c) Photochromism‐modulated UOP for multistage information decryption based on different phenylpyridine/α‐CD solid supramolecules (① 4‐PBA/α‐CD, ② 3‐PBA/α‐CD, ③ ⑥ 4‐PP/α‐CD, ④ 2‐PBA/α‐CD, ⑤ 4‐PBA/β‐CD, ⑦ 4‐PBN/α‐CD).

To verify the application potential of solid 4‐PBA/α‐CD in information security, we fabricated photo‐responsive QR code patterns with two groups: one incorporating solid 4‐PBA/α‐CD as the photochromic functional layer in two corner positioning modules, and the other as an unmodified control (Figure 6a). Upon UV irradiation, the 4‐PBA/α‐CD‐containing positioning modules gradually turned from white to green, a visual change driven by the generation of photogenerated radicals and the emergence of characteristic absorption bands in the visible region, while the control group retained its original black‐and‐white appearance with no discernible color change; subsequent scanning tests demonstrated that the UV‐irradiated functionalized QR code could be successfully recognized by smartphones, whereas the unmodified control failed to be identified due to the absence of effective structural changes in its encoding, thus validating solid 4‐PBA/α‐CD as a feasible photochromic medium for controllable information encryption. Moreover, Figure 6b demonstrated the photopatterning application of the phenylpyridine/α‐CD solid supramolecules enabled by photochromism‐modulated UOP. Two cupcake‐shaped patterns were presented, where the upper row showed the untreated sample, and the lower row showed the sample after photopatterning via selective UV irradiation. Under daylight, the untreated pattern appeared pale and featureless, while the photopatterned pattern exhibited distinct color gradients with blue‐pink hues in the cream and colorful stripes on the wrapper due to photochromic changes induced by UV‐irradiation. Upon UV excitation, both patterns emitted bright cyan‐blue photoluminescence, clearly revealing the full cupcake structure. After the UV lamp was turned off, the untreated pattern showed UOP emission with varying decay rates, where the cream portion retained a strong, long‐lasting afterglow, while the wrapper portion faded rapidly, leaving only the cream outline. In contrast, the photopatterned pattern showed accelerated decay in the wrapper region, resulting in an even more distinct afterglow profile where only the cream part remained visible (Figure S41). This application demonstrates the ability to tailor UOP lifetimes via photochromism for dynamic, stimuli‐responsive photopatterning.

In addition, to demonstrate the advanced multistage anti‐counterfeiting enabled by the interplay between photochromism and photo‐modulated UOP in our supramolecular constructs, we fabricated a seven‐segment display using phenylpyridine/α‐CD supramolecules with tailored photophysical properties (Figure 6c). Under daylight, the unirradiated display showed a blank “8” pattern, while the UV‐irradiated display revealed the first decrypted information “3” via photochromic color changes in specific segments. Upon UV excitation, both displays emitted bright blue UOP to show the transient digit “9”. After the UV lamp was turned off, the UOP decay diverged significantly due to photochromism‐modulated lifetimes, where segments that had undergone photochromism exhibited shortened UOP lifetimes, quenching much faster than their unmodified counterparts. Consequently, at the same time point (e.g., 5 s after UV off), the unirradiated display showed “7” while the UV‐treated display already displayed “1”, achieving time‐resolved dual decryption. This stimuli‐responsive encryption requires both light‐induced photochromism and precise temporal synchronization to unlock complete information, greatly enhancing anti‐counterfeiting security.

## 3 Conclusion

In summary, a facile and green supramolecular assembly strategy was developed to construct photochromic ultralong organic phosphorescence (UOP) materials using phenylpyridine derivatives and α‐cyclodextrin (α‐CD). The rigid microenvironment furnished by α‐CD via host‐guest inclusion and hydrogen bonding not only suppressed non‐radiative decay to stabilize triplet excitons for efficient UOP, but also promoted reversible photoinduced electron transfer to generate persistent radicals, enabling distinct solid‐state photochromism and overcoming the conformational freedom bottleneck of traditional photochromic systems. This dual photo‐functional behavior could be generalized to various phenylpyridine analogs, where substituent electronic and positional effects tailored photophysical performances, validating the versatility of our strategy. Moreover, radical‐mediated photochromism served as a reversible optical switch to dynamically modulate UOP with good cycling stability. This work provides a concise approach to multifunctional photo‐responsive materials, showing promising potential in multistage information encryption and multistate optical patterning.

## Conflicts of Interest

The authors declare no conflicts of interest.

## Supporting information




**Supporting File**: advs76554‐sup‐0001‐SuppMat.docx.

## Data Availability

The data that support the findings of this study are available in the supplementary material of this article.
