# Supplementary material for: Tunable Reversible Photochromic Ultralong Organic Phosphorescence via a Universal Phenylpyridine Noncovalent Assembly Strategy
Source: Adv Sci (Weinh). 2026 Jul 21:e76554. Online ahead of print. doi: 10.1002/advs.76554 (PMC13387033; doi:10.1002/advs.76554)
Supplement: Supplementary file 1 — Supporting File: advs76554‐sup‐0001‐SuppMat.docx. [file ADVS-9999-e76554-s001.docx]

**Tunable Reversible Photochromic Ultralong Organic Phosphorescence via a Universal Phenylpyridine Noncovalent Assembly Strategy**

*Xue Bai,^+[a]^ Renliang Wang,**^+[a^**^]^, Hong-Jin Xue*^[c]^ Zhuofan Jiang,^[a]^ Shuo Yang,^[a]^ Chiming Li,^[a]^ Yanqing Ge,**^[a]^ Caihong Liu,*^[b]^ and Xian-Yin Dai*^[a]^*

[a] X. Bai, R. Wang, Z. Jiang, S. Yang, C. Li, Y. Ge, X. Dai
School of Chemistry and Pharmaceutical Engineering, Shandong First Medical University & Shandong Academy of Medical Sciences, Taian, 271016, China, E-mail: daixianyin@sdfmu.edu.cn

[b] C. Liu
School of Pharmaceutical Sciences & Institute of Materia Medica, Shandong First Medical University & Shandong Academy of Medical Sciences, Jinan, 250117, China, E-mail: chliu@sdfmu.edu.cn

[c] H. Xue
Medical Engineering and Technology Research Center, School of Radiology, Shandong First Medical University & Shandong Academy of Medical Sciences, Jinan, 250117, China, E-mail: hjxue@sdmu.edu.cn

**Supporting Information**

**Table of Content**

**Section A. Experimental Section…………………………………………………………….….….S4**

**Section B. Characterization Data…………………………………………………………….……S5**

**Section C. Reference…………………………………………………………****….……….….…..….S22**

**Section A. Experimental Section**

**Materials and measurements**

All reagents used in the experiment were obtained from commercial suppliers and all solvents were purified in accordance with standard laboratory procedures before use. Photoluminescence spectra, lifetimes were obtained on FS5 instrument (Edinburg Instruments, Livingstone, UK). Fluorescence lifetimes and quantum yields were measured from FLS1000 instrument (Edinburg Instruments, Livingstone, UK) equipped with a picosecond pulsed laser (280 nm-EPLED) as the light source. NMR spectra were obtained via a Bruker AV400 instrument where deuterated water and deuterated dimethyl sulfoxide were obtained from Adamas. Circular dichroism spectra were obtained from JASCO J-810 Spectrophotometer. Rheological measurements were conducted on a Haake Mars40 rheometer (Germany). Electron paramagnetic resonance measurements were performed on a Bruker EMXmicro spectrometer. X-ray diffraction patterns were acquired using a Rigaku SmartLab SE spectrometer (Japan), while Fourier transform infrared spectra were recorded with a Nicolet iS20 spectrometer (Thermo Fisher Scientific, USA).

**Preparation process of phenylpyridine/α-CD solid supramolecules**

The 4-PBA/α-CD supramolecule was prepared via an ultra-facile and green protocol without the need for any chemical additives. In a representative synthesis, α-cyclodextrin (α-CD, 0.578 mmol, 563 mg) and 4-(pyridin-4-yl)benzoic acid (4-PBA, 0.386 mmol, 76.8 mg) were accurately weighed and sequentially added to a 10 mL glass vial, followed by the addition of 4 mL of deionized water. The mixture was subjected to bath sonication at room temperature for 30 min to promote in situ host-guest complexation and hydrogen bonding between the components, and then allowed to stand overnight to form a stable supramolecular assembly. For the fabrication of β-CD- and γ-CD-based aqueous assemblies, the procedure was identical except that α-CD was replaced with an equimolar amount of β-CD or γ-CD, respectively. For assemblies involving other compounds (4-PP, 4-PBN, 3-PBA, 2-PBA), 4-PBA was substituted with an equimolar amount of the target compound, while all other conditions remained unchanged. The resulting aqueous assembly was transferred to a clean Petri dish and placed in a preheated precision oven at 348 K for 12 h to remove the bulk water via thermal dehydration. The sample was then further heated at 373 K for 6 h to eliminate residual moisture, affording a dry, powdery solid supramolecular product.

**Section B. Characterization Data**

**Figure S1.** ^1^H NMR (400 MHz, 298 K) spectra of free 4-PBA recorded in (a) DMSO-d₆ and (b) a 1:1 D₂O/DMSO-d₆ binary solvent system. The incorporation of D₂O only resulted in peak broadening, whereas the chemical shifts remained almost unchanged.

**Figure S2.** Phase solubility diagrams of 4-PBA with α-CD in standard PBS solution at 25 ℃.

**Figure S3.** Circular dichroism spectrum of 4-PBA under ambient condition.


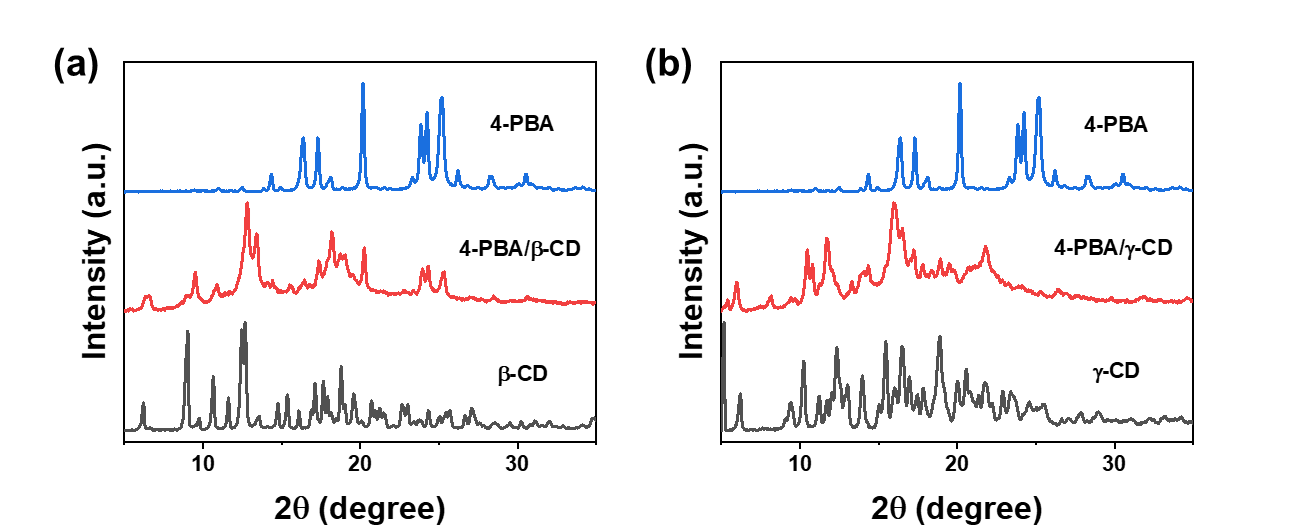


**Figure S4.** Powder X-ray diffraction patterns of (a) 4-PBA, β-CD, and 4-PBA/β-CD, respectively; and (b) 4-PBA, γ-CD, and 4-PBA/γ-CD, respectively.

**Figure S5.** Chemical structure and corresponding high-performance liquid chromatography of 4-PBA.

**Figure S6.** The prompt photoluminescence spectra of 4-PBA solid powder at 298 K.

**Figure S7.** (a) The prompt photoluminescence spectrum and (b) gated emission spectrum (delay 1 ms) of 4-PBA/α-CD, 4-PBA/β-CD, and 4-PBA/γ-CD in aqueous solution.


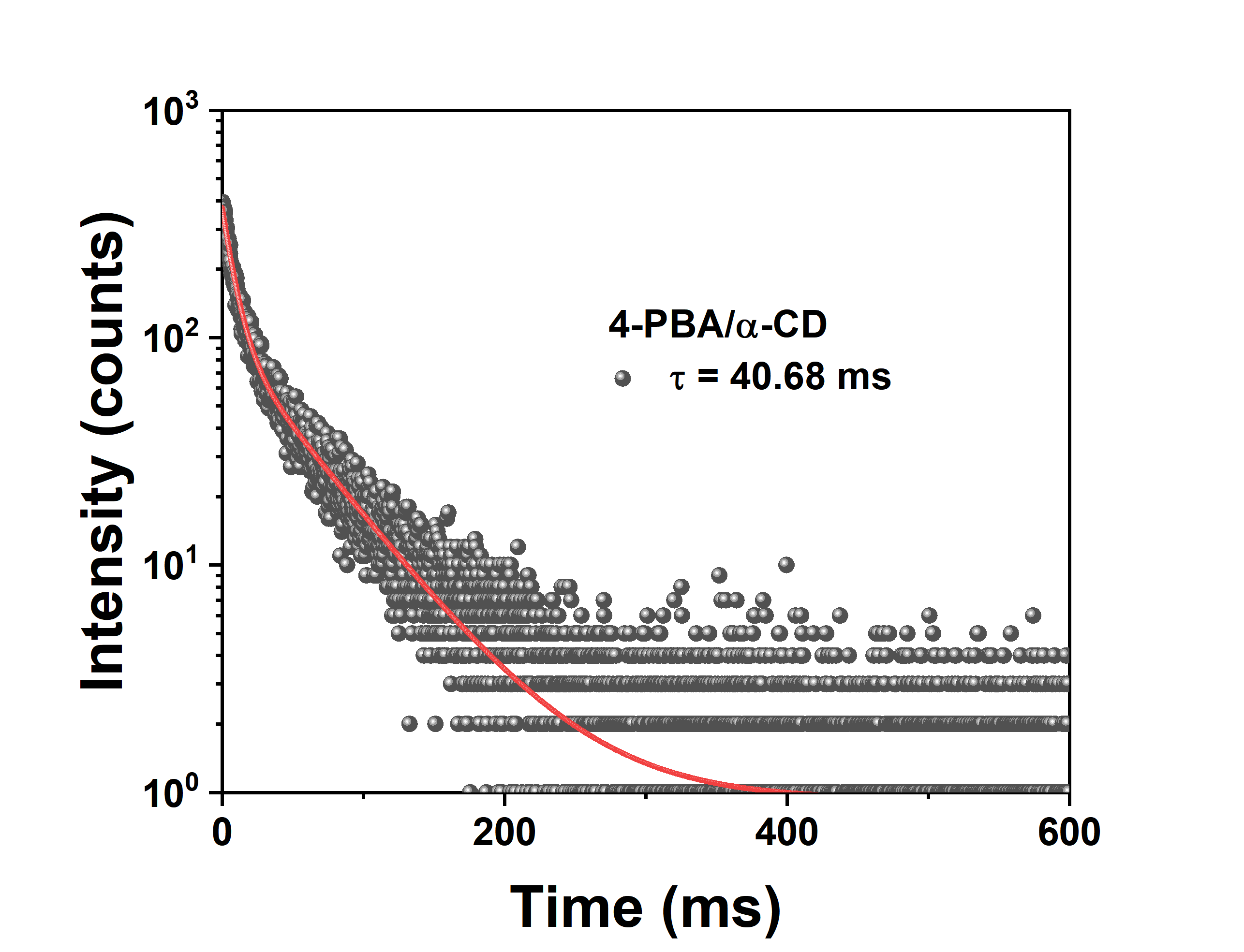


**Figure S8.** Time-resolved photoluminescence decay spectra of 4-PBA/α-CD at 475 nm at 298 K in aqueous solution.


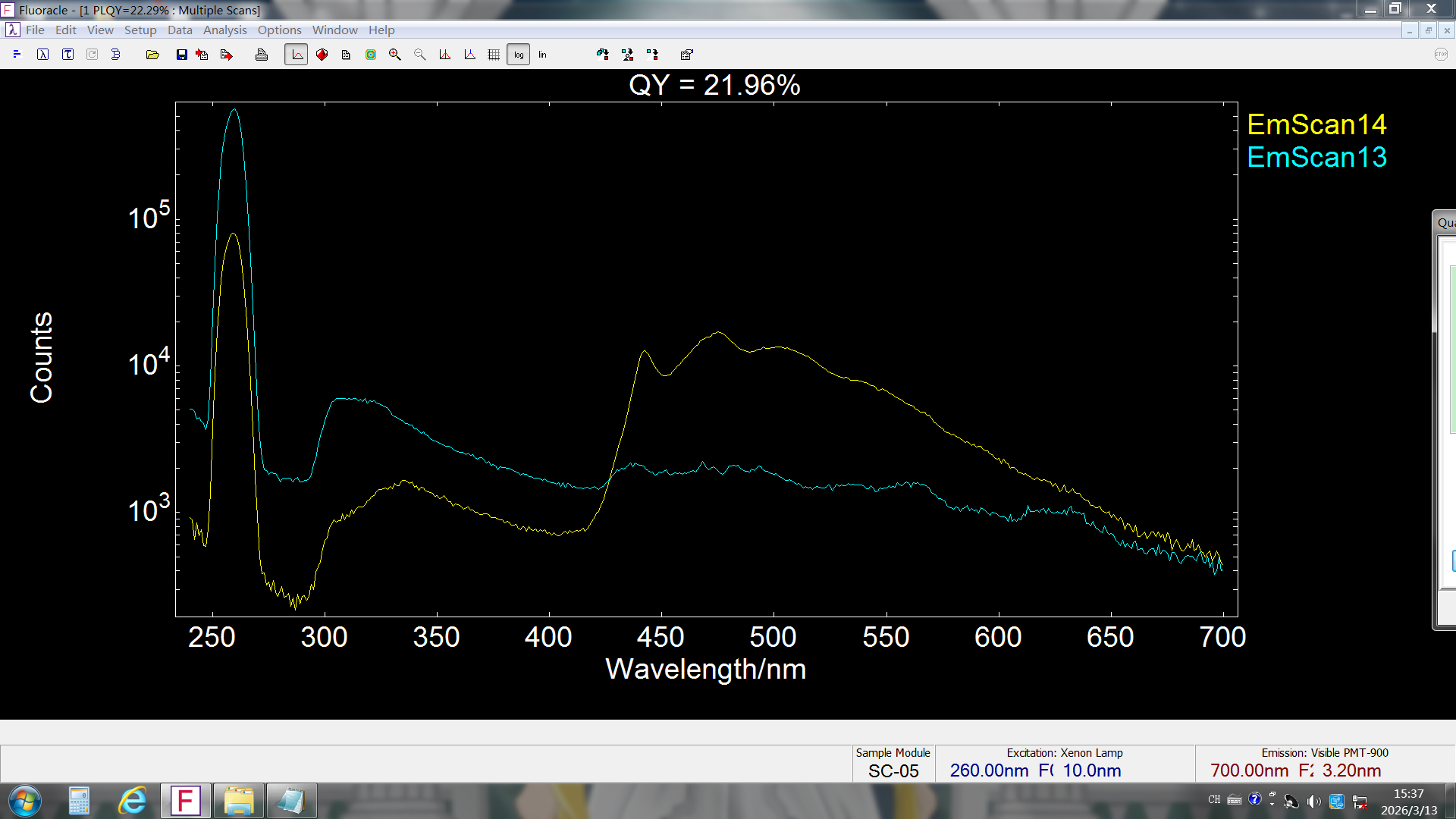


**Figure S9.** Phosphorescence quantum yield of 4-PBA/α-CD supramolecule from 400 nm to 700 nm.

**Figure S10.** The gated emission spectrum (delay 1 ms) of solid 4-PBA.

**Figure S11.** The prompt photoluminescence spectra of solid 4-PBA/β-CD and 4-PBA/γ-CD.

**Figure S12.** Time-resolved photoluminescence decay spectrum of 4-PBA/β-CD at 298 K.

**Figure S13.** PL excitation spectrum of 4-PBA/α-CD before irradiation, after irradiation, and recovery, respectively.

**Figure S14.** FT-IR spectra and powder X-ray diffraction patterns of 4-PBA/α-CD before irradiation, after irradiation, and recovery, respectively.


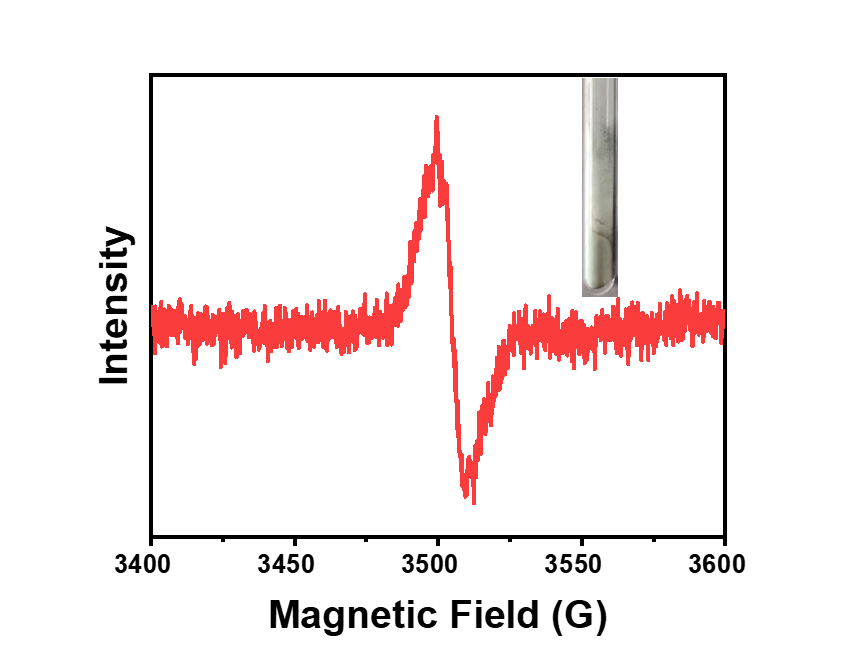


**Figure S15.** Electron paramagnetic resonance (EPR) spectra of 4-PBA/α-CD after 10 min UV irradiation in a glove box under anhydrous and deoxygenated conditions. Inset: The corresponding photograph of the photochromic color of 4-PBA/α-CD after 10 min UV irradiation.


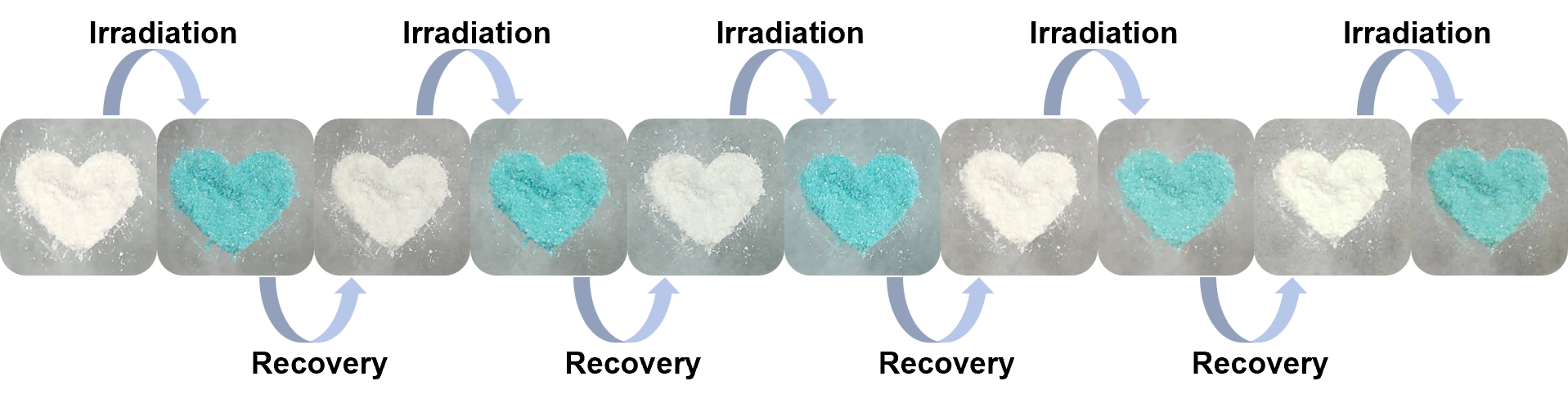


**Figure S16.** Photograph of coloring-fading cycles of 4-PBA/α-CD without obvious color loss after repeated multiple times.


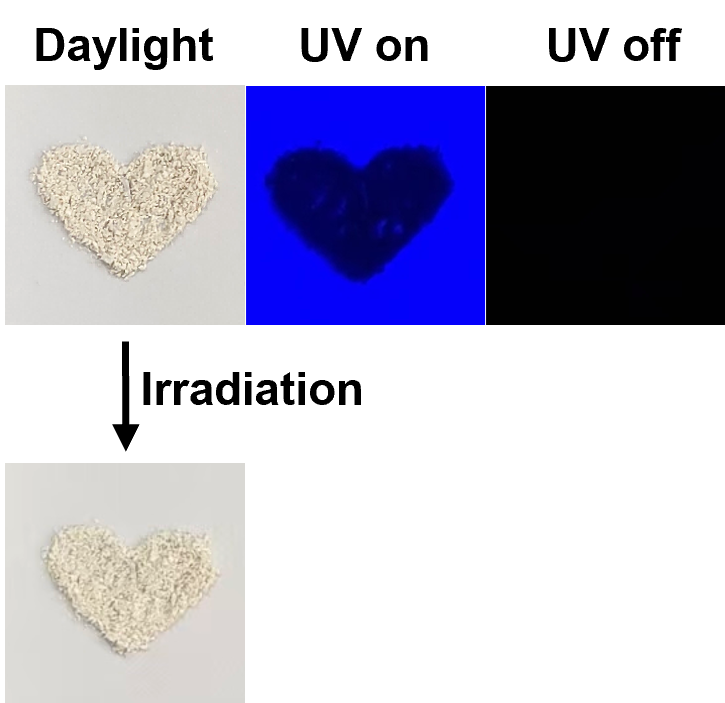


**Figure S17.** Photographs of the visual color of 4-PBA before and after 30 min UV irradiation.

**Figure S18.** EPR profiles of 4-PBA/β-CD and 4-PBA/γ-CD after 24 h recovery.


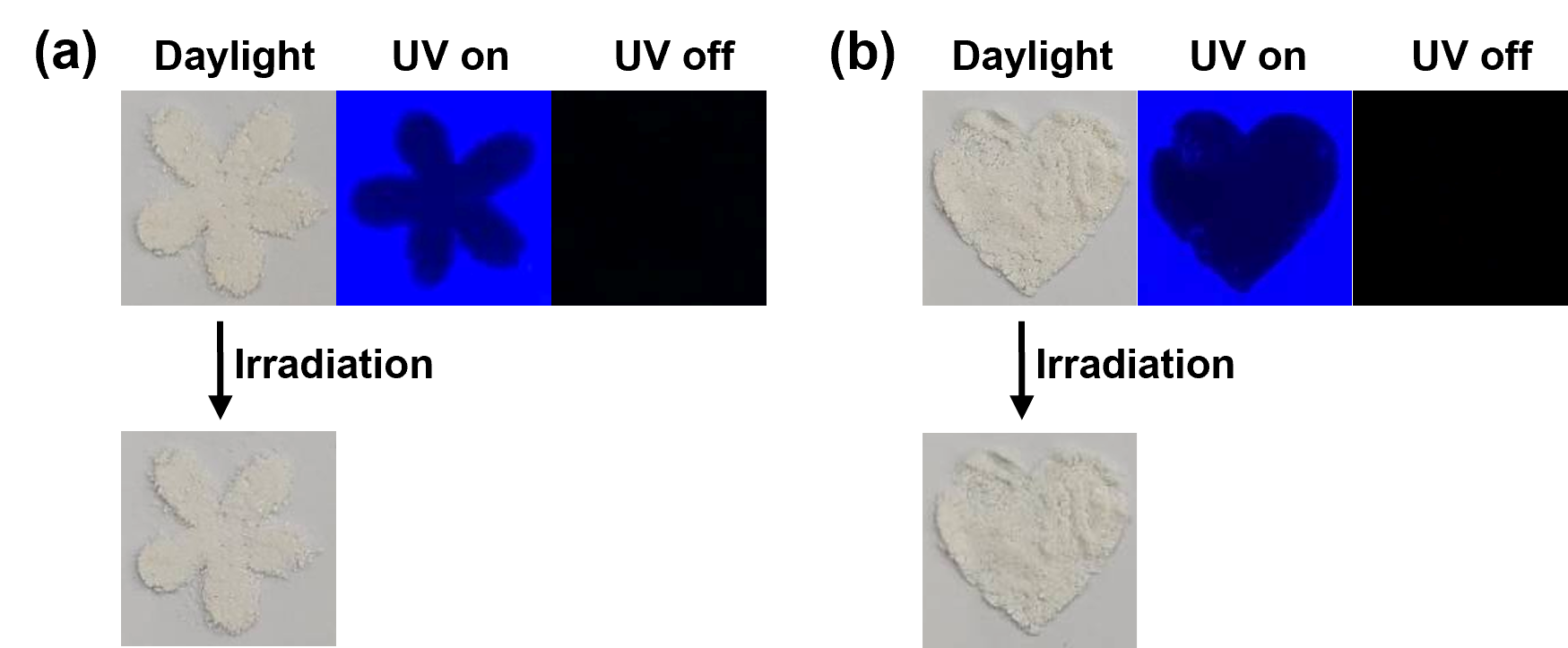


**Figure S19.** Photographs of the visual color of (a) 4-PBA/β-CD and (b) 4-PBA/γ-CD before and after 30 min UV irradiation.


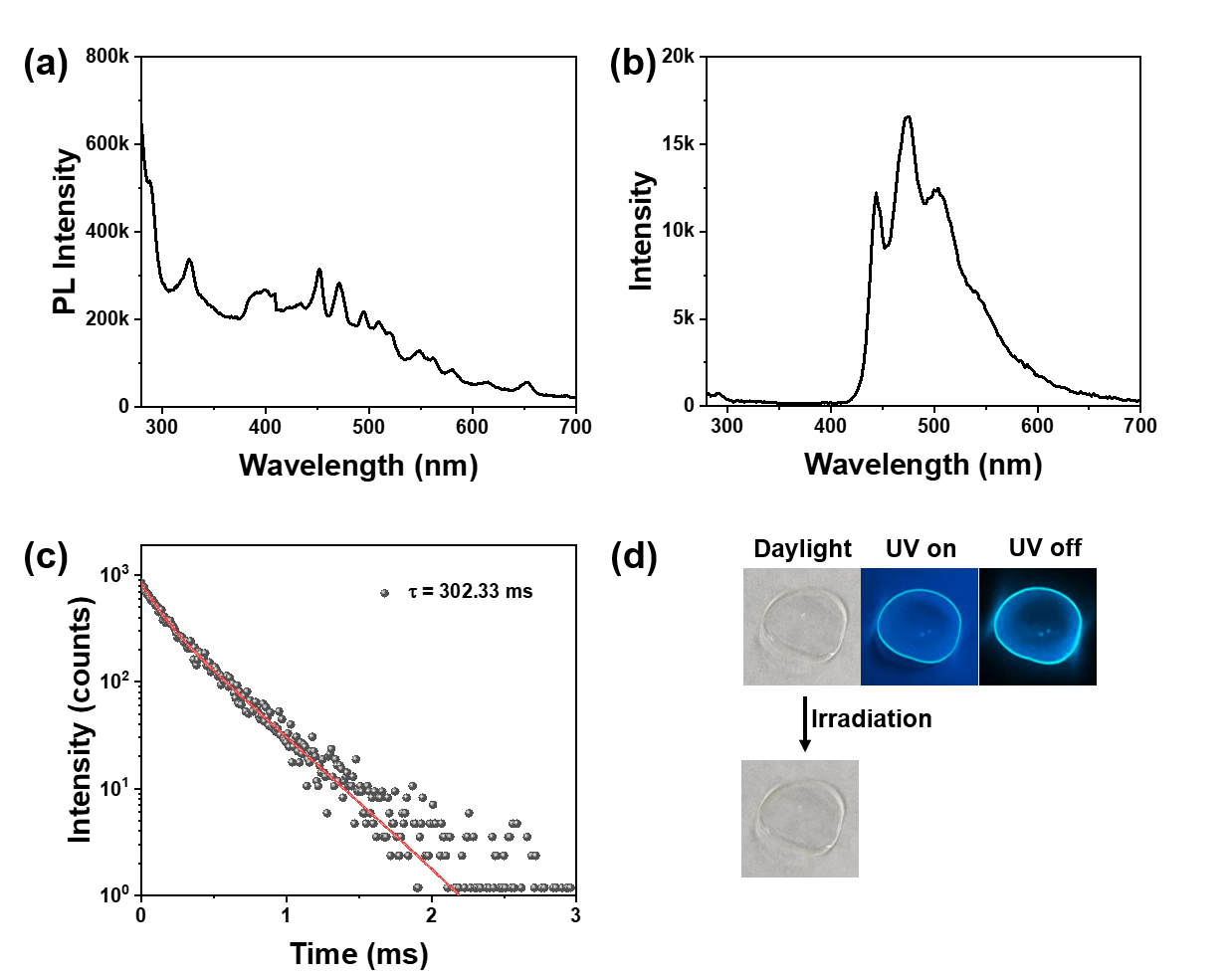


**Figure S20.** (a) The prompt photoluminescence spectra and (b) gated emission spectra (delay 1 ms) of 4-PBA/PVA. (c) Time-resolved photoluminescence decay spectrum of 4-PBA/α-CD at 475 nm at 298 K. (d) Photographs showing the visual color of 4-PBA/PVA prior to and following light irradiation.

**Figure S21.** Changes in (a) photoluminescence intensity and (b) lifetime of 4‑PBA/α‑CD under alternating UV irradiation and recovery conditions.


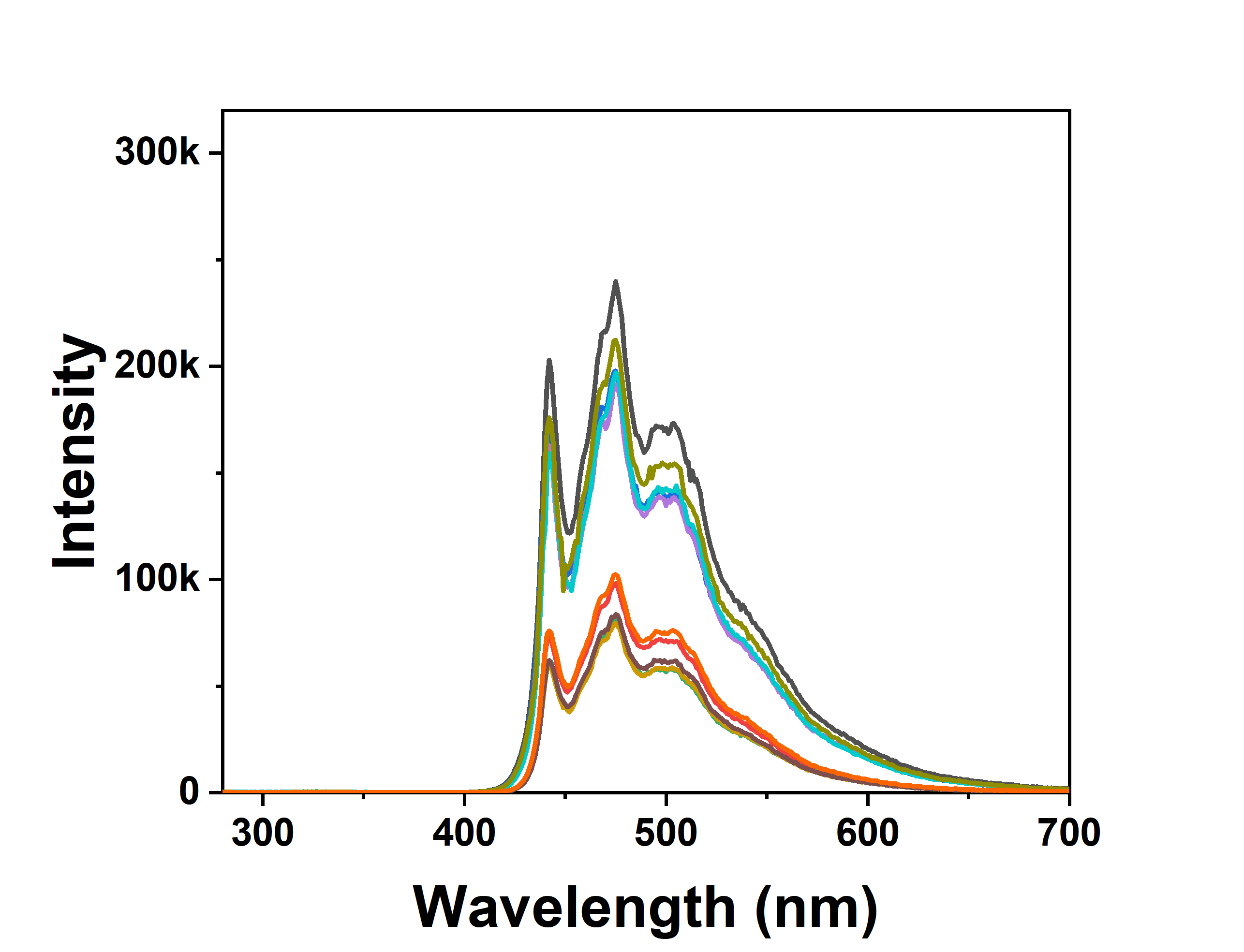


**Figure S22.** Changes in phosphorescence intensity of 4‑PBA/α‑CD under alternating UV irradiation and recovery conditions.

**Figure S23.** (a) The prompt photoluminescence spectra and (b) gated emission spectra (delay 1 ms) of solid 4-PP.

**Figure S24.** (a) The prompt photoluminescence spectra and (b) gated emission spectra (delay 1 ms) of solid 4-PBN.

**Figure S25.** (a) The prompt photoluminescence spectra and (b) gated emission spectra (delay 1 ms) of solid 3-PBA.

**Figure S26.** (a) The prompt photoluminescence spectra and (b) gated emission spectra (delay 1 ms) of solid 2-PBA.

**Figure S27.** (a) The prompt photoluminescence spectra and (b) gated emission spectra (delay 1 ms) of solid 4-PP/α-CD, 4-PP/β-CD, and 4-PP/γ-CD, respectively.

**Figure S28.** (a) The prompt photoluminescence spectra and (b) gated emission spectra (delay 1 ms) of solid 4-PBN/α-CD, 4-PBN/β-CD, and 4-PBN/γ-CD, respectively.

**Figure S29.** Time-resolved photoluminescence decay spectra of solid (a) 4-PP/α-CD and (b) 4-PBN/α-CD at 298 K, respectively.


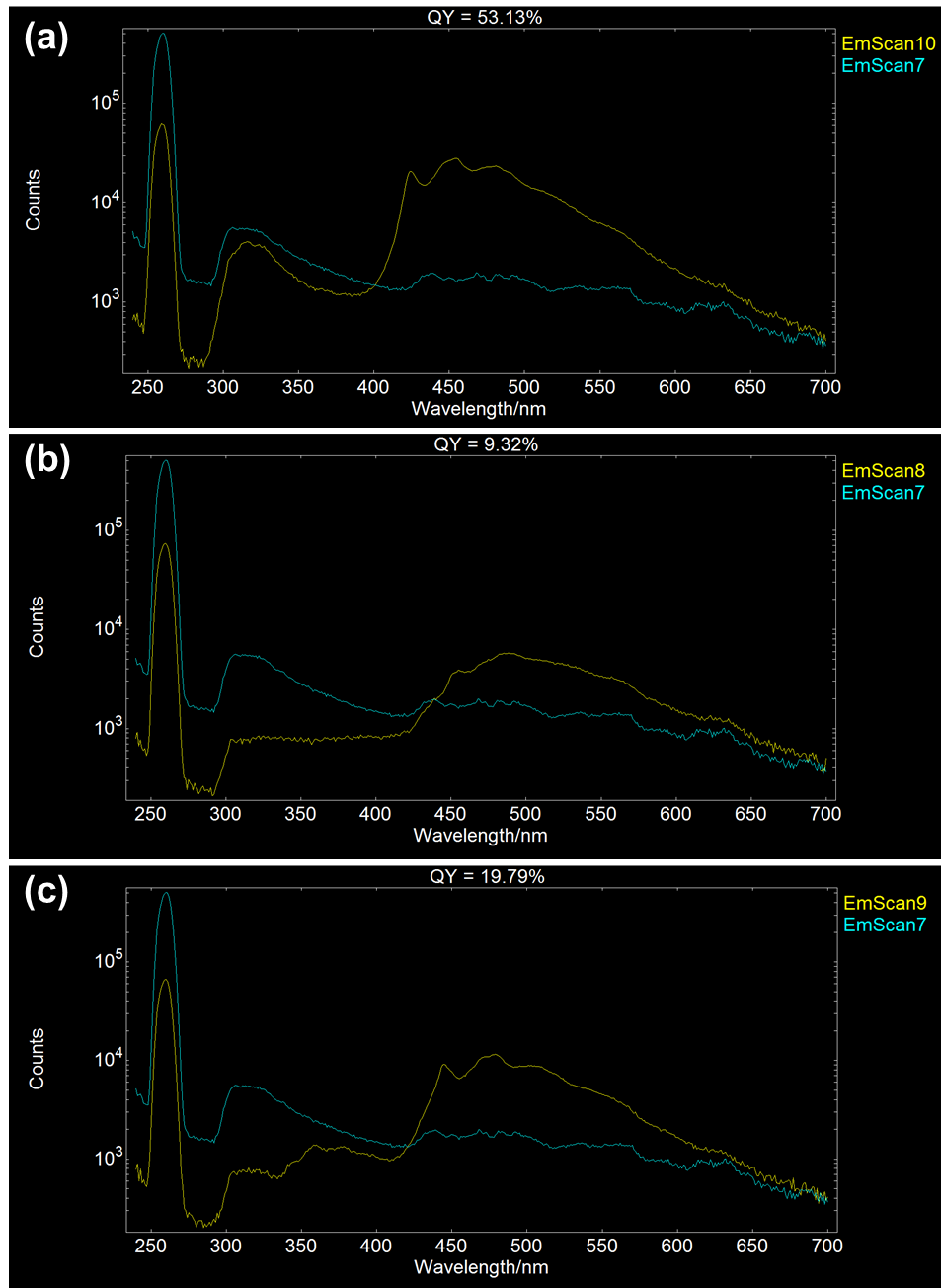


**Figure S30.** Phosphorescence quantum yield of (a) 4-PP/α-CD, (b) 4-PBN/α-CD (c) 2-PBA/α-CD supramolecules from 400 nm to 700 nm.

**Figure S31.** (a) The prompt photoluminescence spectra and (b) gated emission spectra (delay 1 ms) of solid 3-PBA/α-CD, 3-PBA/β-CD, and 3-PBA/γ-CD, respectively.

**Figure S32.** (a) The prompt photoluminescence spectra and (b) gated emission spectra (delay 1 ms) of solid 2-PBA/α-CD, 2-PBA/β-CD, and 2-PBA/γ-CD, respectively.

**Figure S33.** Time-resolved photoluminescence decay spectra of solid (a) 3-PBA/α-CD and (b) 2-PBA/α-CD at 298 K, respectively.


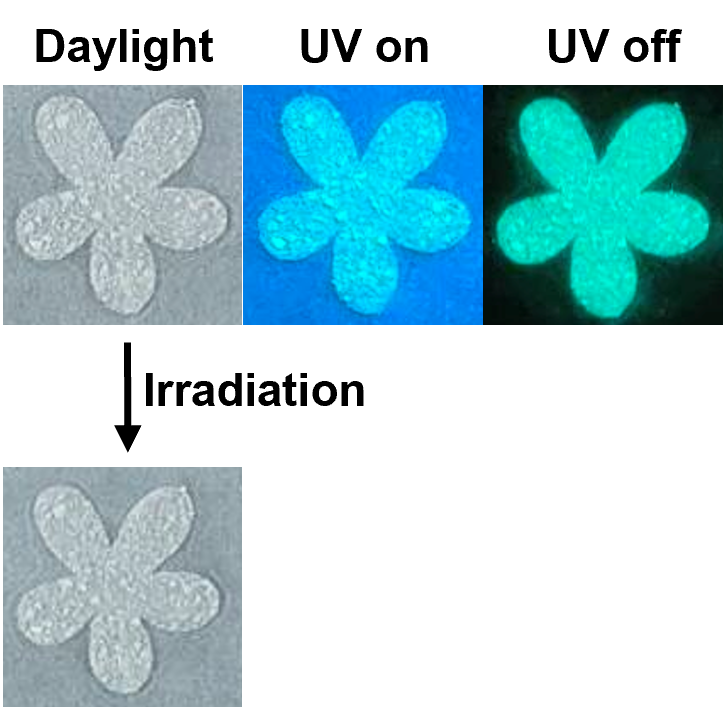


**Figure S34.** Photographs showing the visual color of 3‑PBA/α‑CD prior to and following light irradiation.

**Figure S35.** Electron paramagnetic resonance (EPR) spectra of (a) 2-PBA/α-CD, (b) 4-PBN/α-CD, and (c) 4-PP/α-CD after 10 min UV irradiation in a glove box under anhydrous and deoxygenated conditions.(d) The corresponding photographs of the photochromic color of 2-PBA/α-CD, 4-PBN/α-CD, and 4-PP/α-CD after 10 min UV irradiation.

**Figure S36.** FT-IR spectra of (a) 2-PBA/α-CD, (b) 4-PBN/α-CD, and (c) 4-PP/α-CD before irradiation, after irradiation, and recovery, respectively.

**Figure S37.** Powder X-ray diffraction patterns of (a, b) 2-PBA/α-CD, (c, d) 4-PBN/α-CD, and (e, f) 4-PP/α-CD before irradiation, after irradiation, and recovery, respectively.


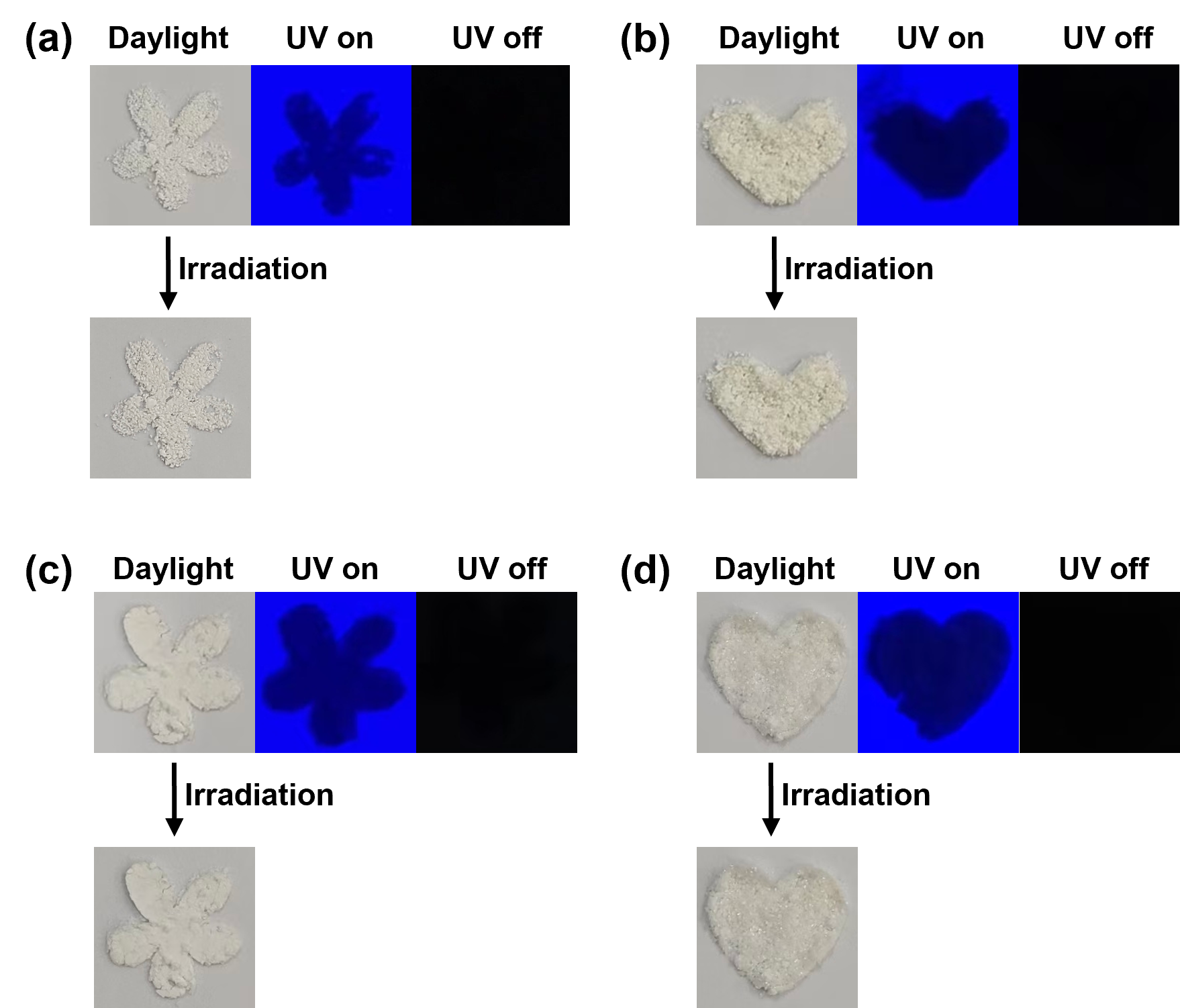


**Figure S38.** Photographs of the visual color of (a) 3-PBA, (b) 2-PBA, (c) 4-PBN, and (d) 4-PP before and after 30 min UV irradiation.

**Figure S39.** UV-vis spectra of 3‑PBA/α‑CD under continuous UV irradiation, with the corresponding EPR profiles (irradiation 0 min, irradiation 10 min, and after 24 h recovery) shown in the insets.

**Figure S40.** Changes in photoluminescence intensity of (a) 2-PBA/α-CD, (b) 4-PBN/α-CD, and (c) 4-PP/α-CD under alternating UV irradiation and recovery conditions.


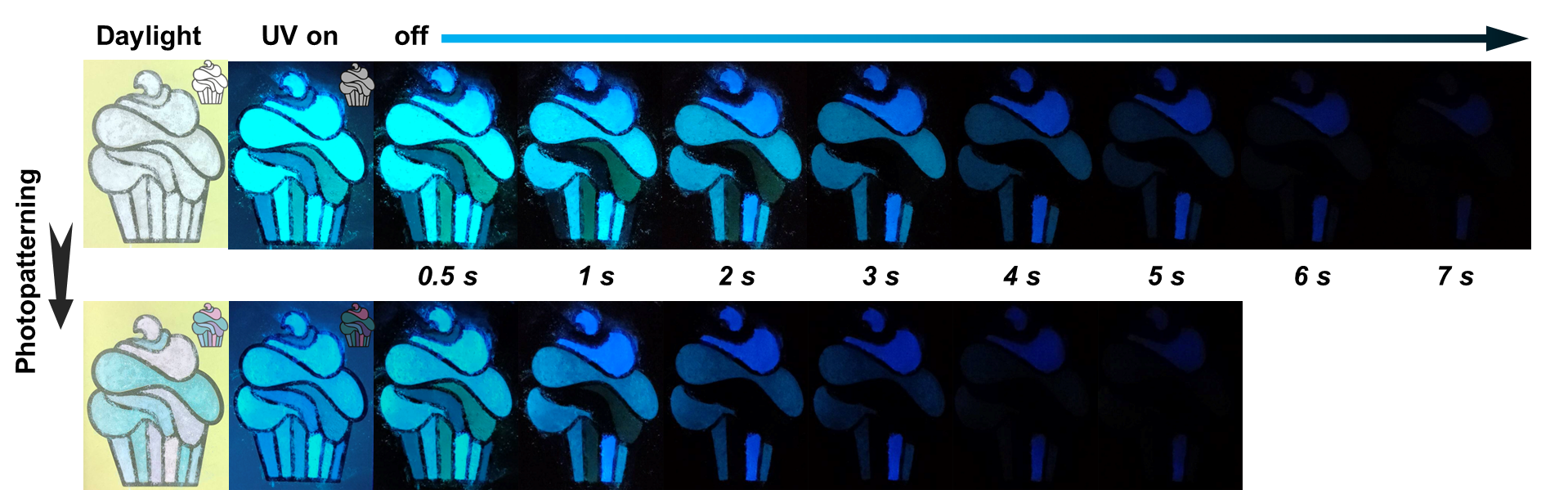


**Figure S41.** Time-resolved photopatterning of cupcake patterns using different phenylpyridine/α-CD solid supramolecules with photochromic and phosphorescent responses.

**Table S1. Summary of recently reported photochromic ultralong phosphorescence materials.**

| Matrix | Guest | Strategy | τ_p_ | Photochromism | Ref. |
| --- | --- | --- | --- | --- | --- |
| Sulfobutylether-β-CD, polyvinyl alcohol | P-CN**/**P-oM**/**P-mM | Copolymerization and assembly | 514 ms, 639 ms, 1056 ms | Dark green, pink | [1] |
| 4,4´-Bipyridine | (Methoxymethyl)triphenylphosphonium chloride and ZnCl_2_ | Grinding, melting, and quenching | 124 ms | Dark blue | [2] |
| Acrylamide | Benzothiadiazole | Copolymerization | 14.3 ms | Dark blue | [3] |
| Polyvinyl alcohol, boric acid | Nonlinear pyridinium compounds | B─O bonding interactions | 0.561 s, 0.247 s, 0.961 s | Dark green, inky, gray | [4] |
| Poly(acrylic acid) | Halogenated (I, Br, and Cl) terpyridine derivatives | Hydrogen-bonding cross-linking | 205 ms | Olive, beige | [5] |
| α-CD | 4-(Pyridin-4-yl)benzoic acid | Aqueous assembly | 769 ms | Cyan blue | This work |
|  | 4-(Pyridin-3-yl)benzoic acid |  | 510 ms | None |  |
|  | 4-(Pyridin-2-yl)benzoic acid |  | 407 ms | Pale purple |  |
|  | 4-Phenylpyridine |  | 957 ms | Light pink |  |
|  | 4-(Pyridin-4-yl)benzonitrile |  | 224 ms | Sky blue |  |

**Section C. Reference**

[1] W.-W. Xu, Y. Chen, X. Xu, Y. Liu, Light and Heat-Driven Flexible Solid Supramolecular Polymer Displaying Phosphorescence and Reversible Photochromism. *Small* **2024**, *20*, 2311087.

[2] F. Nie, D. Yan, Zero-dimensional halide hybrid bulk glass exhibiting reversible photochromic ultralong phosphorescence. *Nat. Commun.* **2024**, *15*, 5519.

[3] B. Ding, H. Gao, C. Wang, X. Ma, Reversible room-temperature phosphorescence in response to light stimulation based on a photochromic copolymer. *Chem. Commun.* **2021**, *57*, 3154-3157.

[4] X. Ma, Y.-Z. Zhu, H.-J. Wang, Y.-J. Mao, P. Gu, S. Zhou, F.-R. Lin, R.-P. Shen, C.-Y. Liu, L.-J. Xu, Engineering Color-Tunable Photochromic Ultra-Long Room Temperature Phosphorescence Polymer Films by Regulating Intramolecular Charge Transfer of Nonlinear Pyridinium Units. *Adv. Funct. Mater.* **2026**, *36*, e21242.

[5] X.-Y. Tian, S.-S. Wu, G.-H. Li, H. Li, B.-H. Wang, R.-N. Chen, Y.-J. Zhang, X.-D. Yu, Radical Photochromism-Induced Phosphorescence Enhancement for Dynamic Anticounterfeiting. *ACS Macro Lett.* **2026**, 10.1021/acsmacrolett.6c00150.
